# General synthesis of inorganic single-walled nanotubes

**DOI:** 10.1038/ncomms9756

**Published:** 2015-10-29

**Authors:** Bing Ni, Huiling Liu, Peng-peng Wang, Jie He, Xun Wang

**Affiliations:** 1 Department of Chemistry, Tsinghua University, Beijing 100084, China

## Abstract

The single-walled nanotube (SWNT) is an interesting nanostructure for fundamental research and potential applications. However, very few inorganic SWNTs are available to date due to the lack of efficient fabrication methods. Here we synthesize four types of SWNT: sulfide; hydroxide; phosphate; and polyoxometalate. Each type of SWNT possesses essentially uniform diameters. Detailed studies illustrate that the formation of SWNTs is initiated by the self-coiling of the corresponding ultrathin nanostructure embryo/building blocks on the base of weak interactions between them, which is not limited to specific compounds or crystal structures. The interactions between building blocks can be modulated by varying the solvents used, thus multi-walled tubes can also be obtained. Our results reveal that the generalized synthesis of inorganic SWNTs can be achieved by the self-coiling of ultrathin building blocks under the proper weak interactions.

## Abstract (web summary)

Single walled nanotubes are promising materials for both fundamental research and advanced applications. Here, the authors develop the synthesis of four types of inorganic single walled nanotube, and show that their formation is initiated by the self-coiling of their ultrathin building blocks.

## Graphical abstract

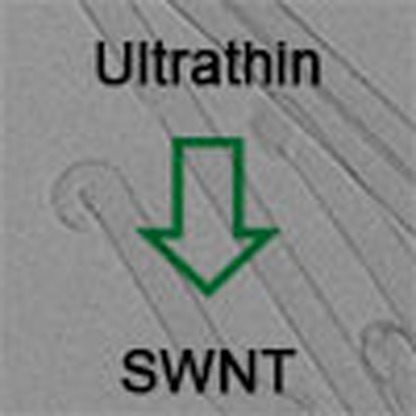

Single-walled nanotubes (SWNTs) are one of the most fascinating materials structural forms, and they have great application potential in fields including catalysis, microfluidic networks, composites, biomaterials and nanodevicesand so on^1^,^2^,^3^,^4^,^5^,^6^. Compared with the numerous SWNT structures of organic molecules (amphiphilic polymers or peptides)^6^,^7^,^8^,^9^, however, there are very few examples of inorganic SWNTs such as carbon and MoS_2_ (refs 1, 2, 10, 11, 12). It is generally believed that layered crystal structures are necessary for the formation of inorganic SWNTs, which has greatly hindered their researches and subsequent applications.

Here we demonstrate the generalized synthesis of inorganic SWNTs: sulfide; hydroxide; phosphate; and polyoxometalate (POM). Detailed studies illustrate that the formation of SWNTs is initiated by the self-coiling of ultrathin building blocks, which is not limited to specific compounds or crystal structures. The unravelling of the initial driving force is essential to the rational design of new materials, and with an understanding of the relationships between building blocks and their interactions, it would be easier to develop more versatile inorganic SWNTs by combining various elements in the periodic table and different bond types. The main difference between inorganic and organic compounds in the formation of SWNTs arises from their inherent difference in structural flexibility associated with the totally different role of weak interactions in their assembly/growth processes. Organic SWNTs could be prepared through a transition from helices to tubes determined by the curvature tolerance ability of the molecule structure^6^,^13^,^14^. For layered inorganic compounds, the flexibility could be achieved by inserting organic compounds into the lattice and forcing the bending of lattice into curved structures like tubes^15^. However, inorganic compounds with non-layered structures are usually rigid and lack flexibility. Very recently, it was found that ultrathin inorganic nanostructures with sizes down to 1 nm can show macromolecules-analogue properties like self-coiling and gelation^16^,^17^. When the size of the inorganics decrease to the molecular scale, weak interactions that can overcome their inherent rigidities play a more important role in their self-assembly process, and as a result more structure flexibility may appear^18^, which provides the possibility of the exploration of inorganic SWNTs in a manner like organic-based SWNTs. Yet there are several different formation mechanisms of different kinds of inorganic tubular structures, despite their synthesis conditions and thickness of wall. For example, the formation of single-walled carbon nanotubes^19^ or MoS_2_ nanotubes^20^ can be described as growth along the axial screw dislocation, screw dislocation with large magnitude of Burgers vectors could lead to tubular structure in the case of ZnO^21^, while ZnO nanorings could also be achieved by epitaxial self-coiling of polar nanobelts^22^, and there are also many template-assisted methods to prepare tubular structures^23^,^24^ and so on. Several kinds of inorganic SWNTs already exist heretofore, including MoS_2_ (ref. 10), BN^25^, WS_2_ (ref. 26), MoO_3_ (ref. 12) and imogolite^27^. However, whether it is the nature of the flexible ultrathin (single) layer that makes it roll into an SWNT, or whether such a structure coincidentally possesses an ultrathin layer, this is an open question which has never been asked. Herein we find that SWNTs, not restricted to layered structures, can be achieved by confining the size of structures. The results demonstrate that ultrathin structures lead to flexibility, and SWNTs could therefore be formed under proper interactions.

## Results

### Synthesis of indium sulfide SWNTs via self-coiling

**Figure 1:**
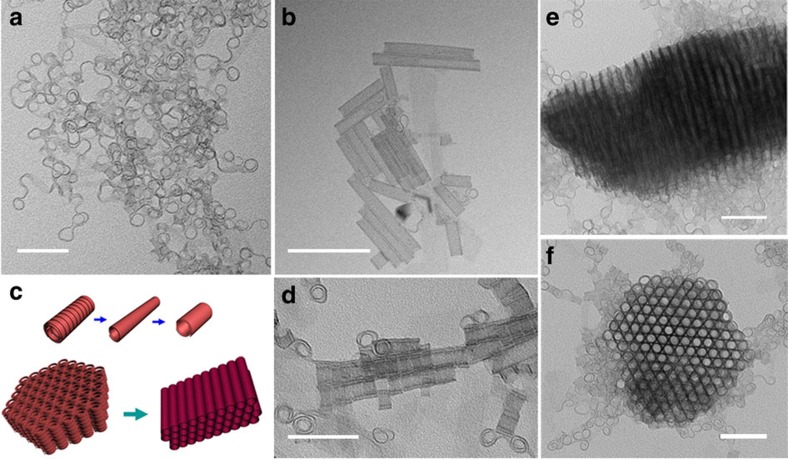
Formation of indium sulfide SWNTs. Nanocoils can be synthesized at 180 °C (**a**), while increasing the temperature to 220 °C would result in SWNTs (**b**). Oriented attachments of nanocoils are found in some TEM images (**d**,**e**, side view; **f**, top view), suggesting a formation mechanism via the coiling of ultrathin nanocoils followed by oriented attachment (**c**). Scale bar: **a**,**d**,**e**,**f**, 50 nm; **b**, 100 nm.

Indium sulfide ultrathin nanocoils were synthesized in our previous study^17^. They showed unexpected self-adjustable self-assembly behaviours in the formation of superlattices and were thus regarded as a mimic of biomolecules in terms of protein crystallization. It is well-accepted that organic SWNTs could be prepared through a transition from helices to tubes, and the main driving force for such systems is the curvature tolerance ability of the molecular structure^6^,^13^,^14^. The more interesting feature is that inorganics may also adopt similar conformations^22^,^28^. Could the macromolecule-like ultrathin nanocoil then convert to an SWNT? Our experimental results provide an exact answer. Increasing the reaction temperature or lengthening the reaction time may help form SWNTs in the solvent system chosen. At 180 °C, only flexible nanocoils (Fig. 1a) could be generated, but SWNTs dominated the products when the temperature increased to 220 °C (Fig. 1b). The detailed analysis indicates that the composition of SWNT is indium sulfide with partial oxygen replacement (Supplementary Figs 1–4). The diameter of the tubes is ∼12 nm and quite uniform, and the thickness of the wall is ∼0.6 nm. Meanwhile, in some transmission electron microscopy (TEM) images, oriented attachments of short-coiled structures can be found (Fig. 1d–f), suggesting a tube-formation mechanism of self-coiling and recrystallization (Fig. 1c, Supplementary Figs 5 and 6). The formation of superlattice structures may further facilitate this process by packing these coils together so that more nanotubes can be found around the supercrystals (Supplementary Fig. 5). It is clear that the self-coiling of ultrathin nanobelts initiates the formation process of indium sulfide SWNTs. Because this self-coiling phenomenon is closely related to the ultrathin size and not limited to specific inorganic compounds, it is safe to propose that inorganic SWNT formation may not require a specific molecule configuration or crystalline structure, but ultrasmall sizes of morphology. Then, the key to the success of this approach would be an efficient system that may steadily generate ultrathin nanostructures.

Fortunately, the current solvent system was found to be quite general in confining the size of inorganic nanostructures within the sub-1 nm region, based on current and previous studies^16^,^17^,^29^,^30^. The general experimental system relies on the combination of a ‘good solvent' and a ‘poor solvent'. Products dissolve well in the ‘good solvent', herein, the ‘good solvent' is an amine (octylamine or oleylamine) or a non-polar solvent like hexane or cyclohexane. The ‘poor solvent' refers to a solvent in which the products dissolve poorly; here ethanol is used. The ‘good solvent' provides a suitable environment for the chemical reaction, while adding the ‘bad solvent' into the system can help increase the supersaturation of products, leading to the formation of small clusters or nuclei^31^. Fine tuning the composition of solvents could enable the production of inorganic nanostructures within the sub-1 nm region.

### Co(OH)_2_ SWNTs and their growth dynamics via the self-coiling process

**Figure 2:**
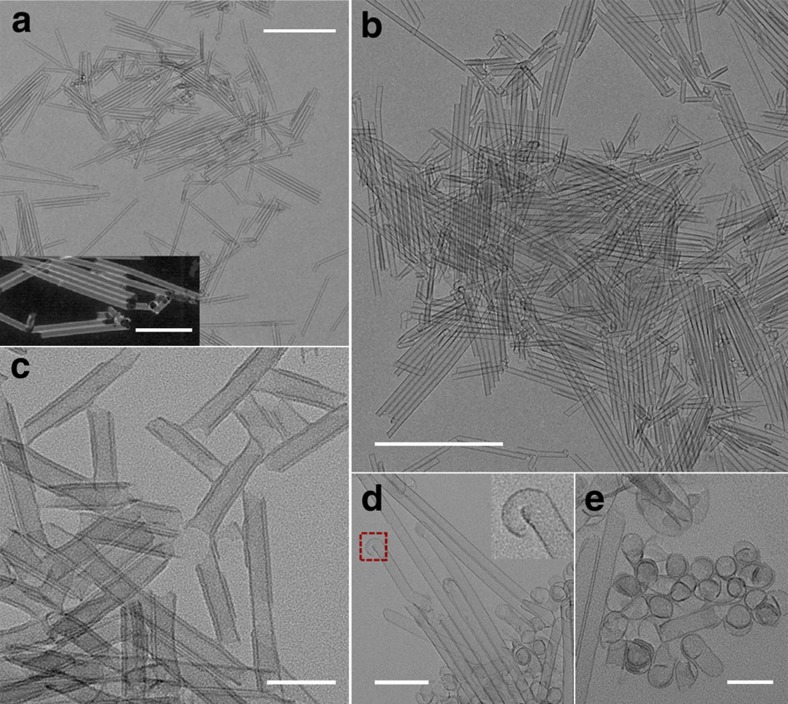
Co(OH)_2_ SWNTs. The as-prepared Co(OH)_2_ tubes are shown here (**a**,**b**, SWNT; **c**, double-layer nanotube). Spiral structures and tubes can coexist by altering the reaction conditions (**d**,**e**). One end of the tube shows a clear transit from a helical structure to a tubular structure by enlarging the area in the red square of (**d**). Scale bar: (**a**) 500 nm, inset, 200 nm; (**b**) 500 nm; (**c**,**e**) 50 nm; (**d**) 100 nm.

**Figure 3:**
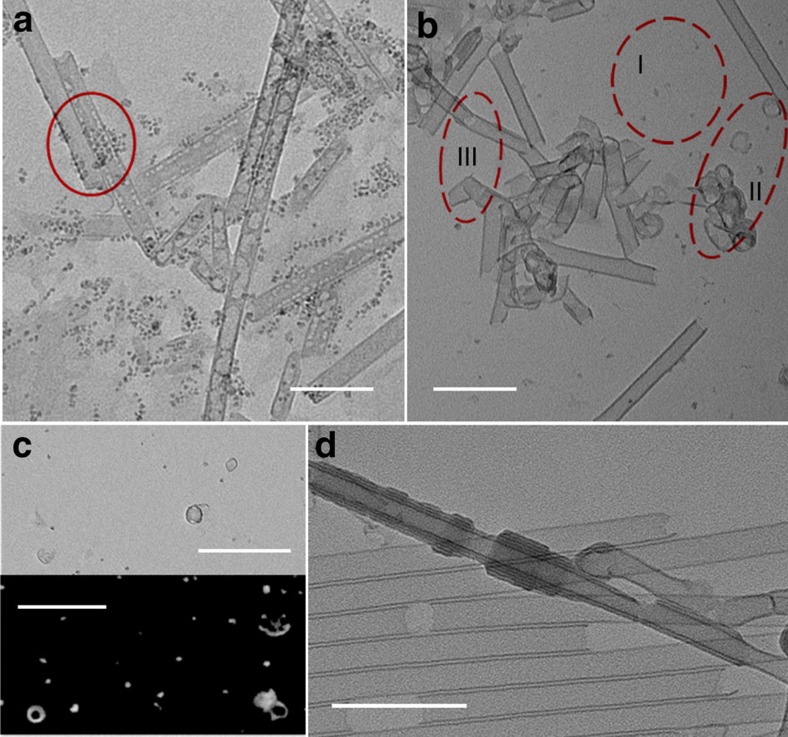
Intermediates and other morphologies in the synthesis of Co(OH)_2_ nanotubes. At 110 °C, only helices and dots (**c**) can be prepared. After totally cooling down the autoclave and then increasing the temperature to 140 °C, nanotubes with holes in the wall can be produced (**a**). The ring-like structures totally disappears, suggesting that they might be intermediates. The red circle of **a** shows some small dots embedded on the wall of the tubes, suggesting that the small dots cannot reconstruct to form walls and thus small dots are not intermediates. The rapid cooling of the autoclave by flushing cold water can furthest maintain the original features of the on-going reaction system and hint at the intermediates (**b**). Comparing the TEM image with corresponding sample prepared by naturally cooling down in air, the dots in area I are expected to be the aggregate of ultrathin intermediates. We may expect that the well-packing of strands in area II could lead to the ring-like structure in **c**, so that such coiling strands might be the intermediates. The interlaced tubes in area III shed light on the termination mechanism of tube growth. (**d**) The possibilities of preparing nanotubes with more layers. Scale bar: (**a**,**b**,**d**) 100 nm; (**c**) 200 nm, inset, 100 nm.

Another example, Co(OH)_2_, was selected to carefully study the SWNT formation. After a precursor reaction in a solvent system similar to that of indium sulfide SWNTs, cobalt hydroxide SWNTs were formed. The average length varied with the reaction time while the diameter remained uniform at ∼20 nm and the wall thickness remained at about 1 nm, similar to the distance of interlayer spacing in α-Co(OH)_2_. By carefully comparing the X-ray diffraction pattern (Supplementary Fig. 7) of our product with that reported in the literature^32^, we found that the (003) diffraction peak of α-Co(OH)_2_, the strongest peak and characteristic of the layered structure, disappeared, meaning that the layered stacking of Co(OH)_2_ was lost. Double-layer or multilayer nanotubes could be achieved by introducing ethanol into the reaction system. Small angle X-ray diffraction indicates a broadening peak with distance about 3.5 nm in the case of DWNT (Supplementary Fig. 8). By checking the TEM image, we can find that the distance between different layers is about 3.5 nm, and their stacking is not strictly uniform, which may broaden the peak. The structure of DWNT might be similar with the case of VO_*x*_ nanotubes^15^, in which the curvature is caused by inserting amine molecular. However, considering the structure of SWNT here, and the phenomena that ultrathin structure could lead to flexibility, it is safer to assume that the octylamine is adsorbed on the backbone, but not insert into backbone. TEM images of as-prepared nanotubes are shown in Fig. 2 (a, single layer; b, double layer; Supplementary Fig. 9, double layer and multilayer) and Fig. 3d. By slightly altering the reaction conditions, SWNTs and helical structures can coexist (Fig. 2d,e). The helical structures in Fig. 2e are actually spiral ultrathin nanosheets with overgrown edges, we have studied the formation mechanism of such structures in our previous study^30^. In that report, we demonstrated the formation of CoFe, CoNi and NiFe bimetallic hydroxides in similar conditions. However, we did not observe the transition from helical structures to tubular structures in that report. Here a clear transformation from helical structure to tubular structure could be observed in Fig. 2d.

To further investigate the growth mechanism, we carried out a stepwise heating process (normal conditions for the synthesis of SWNTs: 140 °C, 8 h) to determine the possible intermediates. The reactant was heated at 110 °C for 8 h and then naturally cooled to room temperature in air; then the temperature was further increased to 140 °C for another 8 h. The results show that only dots and ring-like structures were generated at 110 °C, which means that the tubes cannot grow at relatively low temperatures (Fig. 3c, Supplementary Fig. 10), while the final products of the second step were a mixture of dots (1.2–5 nm), sheets and SWNTs with holes in the walls (Fig. 3a). Carefully analysing Fig. 3a, we find that it is the small dots that occupy the positions of the holes. This means the already-formed dots can grow on the SWNTs, but cannot reconstruct into walls. It would be more reasonable to see these dots simply as the aggregates of intermediates. Because they cannot grow on the tube as intermediates, a smaller size of intermediates, or ultrasmall dots, must exist. This is easy to understand because large sizes can grow from smaller sizes.

Another aspect observed in the stepwise heating process is that the ring-like structures generated in 110 °C reaction disappear at 140 °C. Do they dissolve or are they the intermediates? Flowing cold water can cool the autoclave from the reaction temperature to room temperature in <5 min, so immediately fast cooling the autoclave and washing the products would most closely maintain the original features of the on-going reaction system. Figure 3b is the TEM image of the rapid cooling result of the 4 h specimen (140 °C). The dots in area I and the open ring in area II are not seen in the unaffected cooling specimen (naturally cooling down the autoclave in air, Supplementary Fig. 11c). Considering the sizes of the particles, the dots are more likely to be the aggregate of intermediates. Rings can be considered as ‘ultra-short' nanotubes. Only by the elongation of ‘ultra-short' nanotubes, can long nanotubes be obtained, and this is another way of saying that the rings are the intermediates. The tangled helices in area II suggest the self-folding of flexible helices. We may also expect that the well-packing of strands in area II could lead to the ring-like structure in Fig. 3c, so that such coiling strands might be the intermediates, and the ring-like structures may be formed from helices. On the basis of our experimental results, the ultrathin helices found in area II and the ultrasmall dots widely existing in the reaction process can be regarded as intermediates. To distinguish between the helices and the ultrasmall dots, we call the non-aggregated ultrasmall dots monomers and the helices initiators in the following part.

**Figure 4:**
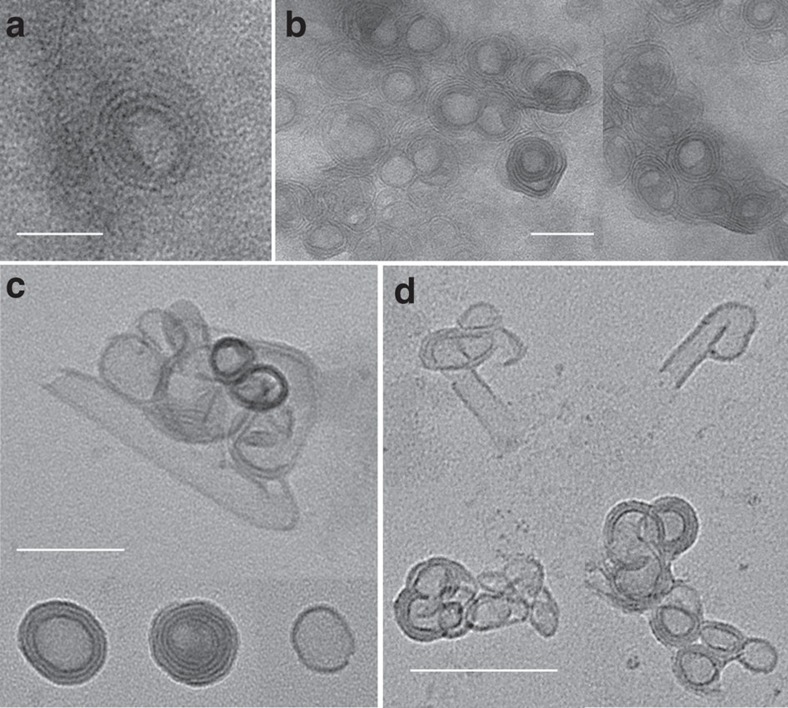
Products produced by fast cooling of autoclaves in incipient period of reactions. At 10 min (**a**), a spiral structure (not concentric rings) embedded in background could be observed. Elongating the reaction time to 20 min (**b**), more spiral structures with even more layers could be seen (left and right panel are acquired from two individual TEM images). At 30 min (**c**), several morphologies could be observed (four structures are acquired from two individual TEM images), such as ring, concentric rings (three layers), spiral structures and nascent tube could be formed. It is clearly that one end of the nascent tube is open while the other end is closed. At 40 min (**d**), more tubes in which one end is open while the other end is closed could be formed. There seems to be a clockwise transit from lower right to up right corner. The tube is protruding from spiral structures which are out of round. (four structures are acquired from one TEM image)This might be one of the experimental evidences which shows unidirectional growth of tube. It is still worth mentioning that fast cooling may maintain features of on-going reactions, but they might not be exactly the same with the situation in on-going reactions. Scale bar: (**a**) 25 nm; (**b**,**c**) 50 nm; (**d**) 100 nm.

The structures of initiators were further studied by fast cooling in the incipient period of reaction (Fig. 4). When the temperature of the oven reached 140 °C, the timing began. We took autoclaves out of the oven at different times and put them into flowing water. At 10 min, a spiral structure (not concentric rings) embedded in the background could be observed. Elongating the reaction time to 20 min, more spiral structures with even more layers could be perceived. At 30 min, ring, concentric rings (three layers), spiral structures and nascent tubes could be formed. It is clearly that one end of the nascent tube is open while the other end is close. At 40 min, more tubes in which one end is open while the other end is close could be formed. This might be one of the experimental evidences, which shows unidirectional growth of tube. It was found that short tubes were usually protruding from spiral structures that are out of round, on one hand, suggesting that there must be a transition from helical structures to tubes, on the other hand, rendering one end close and the other end open.

Once the presence of the monomers and initiators was confirmed, the interactions between these nanobuilding blocks should be investigated as the next key parameter that governs the subsequent growth process of the SWNTs. In our previous study, the interaction strength could influence the morphology of the self-assembled MoS_2_ tubes^29^. A proper interaction strength between the monomers would make a flexible self-assembled structure^33^, that may roll into a helical structure under an optimized interaction strength. In this case, the interactions between monomers may be the static electric force, 
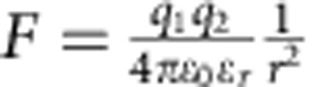
, Keesom force, 
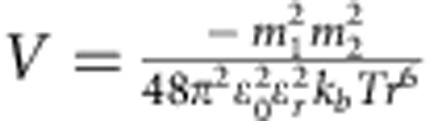
, Debye force, 
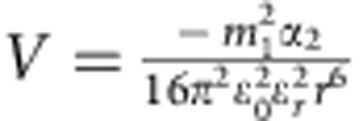
, or London dispersion force, 
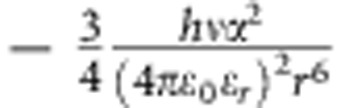
, where *q*=charge, *m*=charge per length, *ɛ*_0_=permittivity of free space, ɛ_*r*_=dielectric constant of surrounding material, *T*=temperature, *k*_*b*_=Boltzmann constant, *r*=distance between molecules, and*α*_2_=polarizability. For all these interactions, the strengths have inverse relationships with the dielectric constant (*ɛ*) of the solvents involved^34^. Hexane is a non-polar solvent and possesses an ɛ equal to 1.9, and we also selected other non-polar solvents like cyclohexane (*ɛ*=2.1), octane (*ɛ*=1.9), decane (*ɛ*=2.4), dodecane (*ɛ*=2.1) and toluene (*ɛ*=2.4) to serve as medium^35^. Despite their great differences in boiling point, viscosity and other physical/chemical property, they all produce SWNTs with similar diameter as the product (Supplementary Fig. 12) under similar conditions. However, solvent with a significantly different dielectric constant, like chloroform (*ɛ*=4.8), could not produce SWNTs as products.

The hypothesis that the interactions between building blocks may greatly influence the formation of SWNTs can be further supported by the formation of double-walled nanotubes. If the interaction is weakened while the other conditions are maintained, then the distance between monomers would increase. As a result, the self-assembling helical structures, or tube growth initiators, may be looser, enabling more layers of the assembling structure to be generated, so that a stable multilayer structure may be formed as the final products. Guided by this perspective, we introduced ethanol (*ɛ*=25.3) into the system to increase the dielectric constant and decrease the interactions, and stable double-layer nanotubes and multilayer nanotubes were obtained (Fig. 2b, Supplementary Fig. 9). We can also occasionally achieve tubes with even more layers (Fig. 3d), suggesting the possibility of preparing nanotubes with more layers. These experiments suggest that the interactions between the monomers are vital and tunable. Hence, proper interactions could be modulated by simply choosing a proper solvent system.

**Figure 5:**
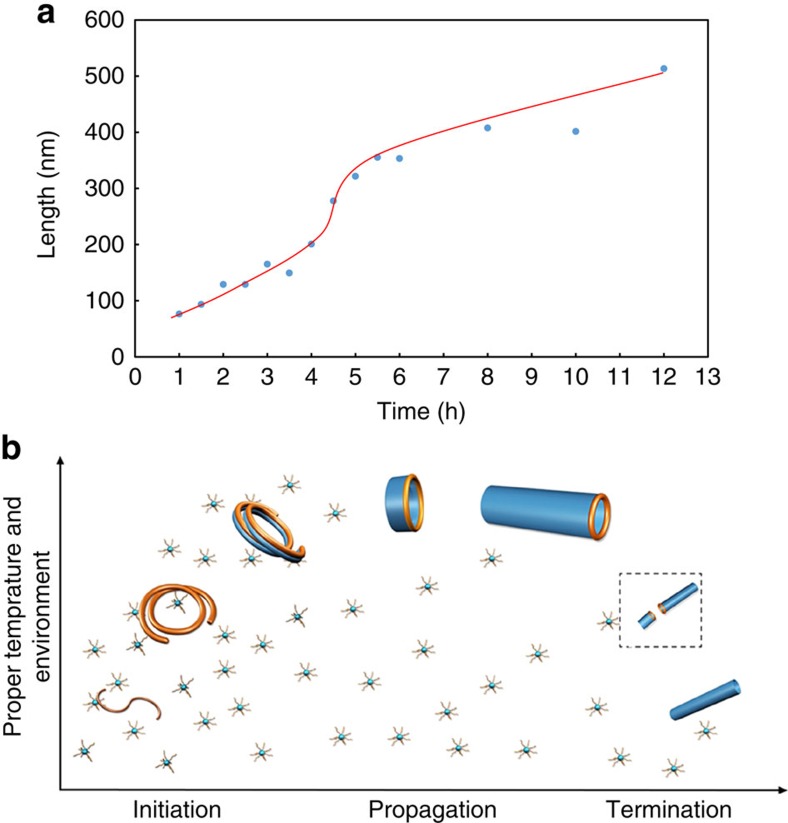
Kinetics of SWNT formation. The length–time graph was plotted (**a**) by using data in Table 1, with the S-shaped line indicating an autoacceleration of the growth rate. The proposed growth mechanism is drawn in **b**. The tube growth initiates with the flexible helical structure formation, and the deposition of monomers onto the densely packed helix drives the formation into the propagation stage. The two ends of tubes are different, and the monomers are more likely to deposit on the open end. The termination of growth is caused by the combination of two growing tubes.

We move forward to the analysis of growth kinetic by considering many experimental phenomena here. The morphological evolution of SWNT proceeded from spiral structures to multilayer nanotubes, some of which interleaved at the end, and finally to SWNT (Supplementary Figs 11 and 13). The evolution from multilayer to single-layer might occur through the sacrifice of high-energy layers as time goes on, as different layers possess different surface strains and energies^21^. We then plot a tube length–time graph (Fig. 5a). In the graph, there is an autoacceleration of tube growth during the elongation of the tubes(see in Methods), as the tubes undergo a linear growth in the first 4 h, and then an autoacceleration happens from 4 to 6 h, following which the growth rate decreases. Meanwhile the polydispersity indexes (PDIs) of the SWNTs are around 1.5 (Table 1, Supplementary Fig. 14). The autoacceleration effect and PDI are reproducible (Supplementary Fig. 15, Supplementary Table 1).

Taking all these phenomena and aforementioned structures in to account, we proposed a mechanism drawn in Fig. 5b. To be concise, we divide the process into three stages, namely initiation, propagation and termination (Fig. 5c). In the initiation stage, monomers self-assemble and roll into helical structures to form initiators. Crystallization would influence the interaction with monomers and drive the growth on only one side of the SWNT^36^,^37^. Here the helices are not symmetrical structures, as the two ends of the helices may be entities at different times, so their interactions with monomers would be different. Altogether with the structures observed at the incipient 40 min of reaction, it would be reasonable to infer that the active site, at where the monomers could deposit, may reside on only one side of the SWNT. The tubes in Fig. 4 might be a direct evidence of such unidirectional growth. As for the termination, two growing sites of different tubes that fused together would terminate the SWNT growth and make the two growing tubes into one tube that cannot grow further. The termination could be caused either by a random reaction between growing tubes with active sites or by cooling down the reaction, considering that if the temperature decreases, the tubes cannot grow. Some tubes interleaved at the end in area III (Fig. 3b), supporting a combination type of termination. Another piece of direct evidence for a combination type termination is that SWNTs prepared by unaffected cooling (∼1 h to cool down to room temperature, but ∼10 min to decrease below 110 °C, under which temperature the tube cannot form) are much longer than those prepared by fast cooling correspondently (Supplementary Table 2). Due to the combination type termination, when the nanotubes grow longer, it would be more difficult to adjust their active sites' orientations to fuse together and terminate the growth. Hence, the termination would slow down as the tube lengthens, resulting in an autoacceleration phenomenon of growth. After that the concentration of monomers decreases and the growth rate slows down again.

### Phosphate SWNTs

**Figure 6:**
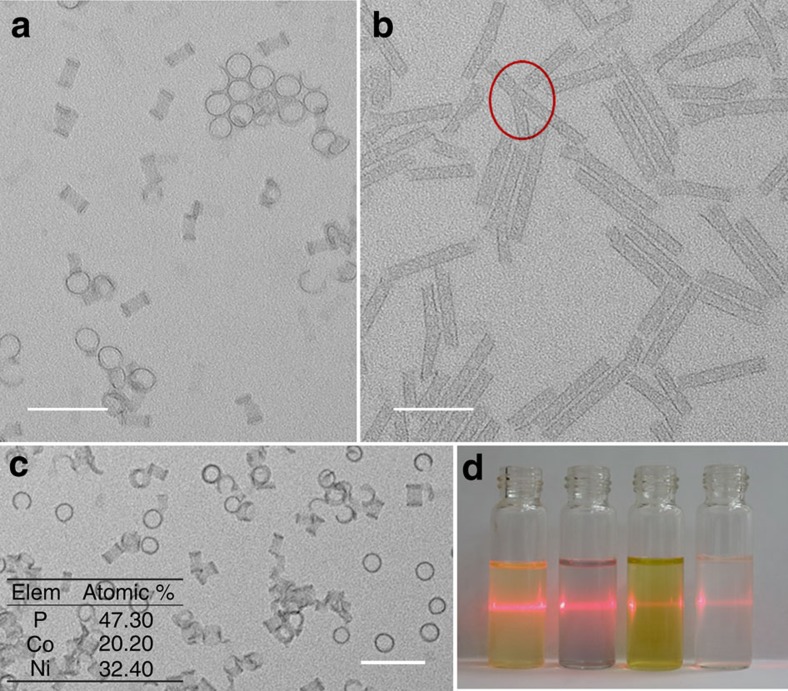
Phosphate SWNTs. In the case of phosphate, nanorings (**a**) and SWNTs (**b**) can be synthesized. A Y-shaped structure can be seen in the red cycle, indicating the possibility of fact that three tubes may fuse together in the termination stage of growth. The effective doping of nickel phosphate nanorings (tubes) could be achieved by simply introducing cobalt or iron ions. Here **c** shows the doping of cobalt into nickel nanorings. (**d**) The optical image of the Tyndall effect of these four types of SWNTs (indium sulfide, cobalt hydroxide, nickel phosphate and POM from left to right), suggesting the good solubilities of these SWNTs. Scale bar, 50 nm.

This is certainly not an isolated case for SWNT formation. In addition to the above indium sulfide and cobalt hydroxide we further expand this strategy to the synthesis of nickel phosphate SWNTs (Fig. 6b, Supplementary Fig. 16). The diameter of the SWNTs obtained is ∼6 nm, the thickness of the wall is ∼0.6 nm and the lengths are ≲50 nm, much shorter than the aforementioned SWNTs. By slightly altering the reaction conditions, we can achieve nanorings as the final products (Fig. 6a). Nanorings can be seen as short nanotubes, but could also be considered densely packed helices. The SWNT might be initiated by the formation of a helical structure under proper environment like in the case of Co(OH)_2_. Here the growth process may also follow the proposed termination mechanism. This can be verified by the observation of the fusing of three reacting tubes to terminate the growth, which produces a Y-shaped tube as shown in the red circle of Fig. 6b, because it would be easier for three short reacting tubes to fuse together to terminate the growth. The synthetic protocol here is quite versatile. Following the same procedure, effective doping can be easily achieved in nickel phosphate SWNTs. For example, iron and cobalt ions that have the same charge and similar radius to nickel ions can be doped into the nanorings or nanotubes (Fig. 6c, Supplementary Figs 17–21), so that the compositions can be easily tuned. This suggests a potential way to design newly advanced SWNTs with different properties by modulating their composition.

### POM SWNTs

**Figure 7:**
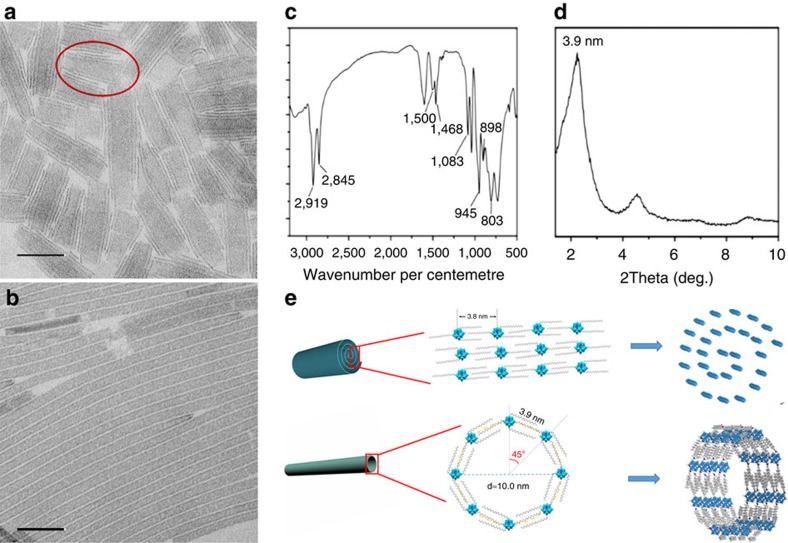
POM SWNTs. Increasing the temperature or elongating the reaction time would convert the POM nanorolls (**a**) to SWNTs (**b**). Scale bar, 50 nm. The roll in the red circle shows an unequal number of ridges, indicating that it is indeed a roll but not a multi-wall tube. In the FT-infrared data, the peaks at 803 cm^−1^ and 898 cm^−1^ are assigned to the O–W–O antisymmetric stretching vibration modes of the bridging oxygen. The peak at 945 cm^−1^ corresponds to the W=O stretching mode of terminal oxygen. The peak at 1,083 cm^−1^ is ascribed to the P–O stretching mode. The peaks at 2,919 cm^−1^ and 2,845 cm^−1^ correspond to the CH_2_ and CH_3_ stretching vibrations and the peaks at 1,500 and 1,468 cm^−1^ correspond to the N−H mode, suggesting that the amine in the precursor exists in SWNTs. (**d**) SAXRD of POM nanotubes indicates a periodic distance of 3.9 nm. The infrared and SAXRD spectra of nanorolls show similar results to the nanotubes. Taking all these results into account, the structures of the nanorolls and SWNTs are depicted in (**e**). The cross-sectional perimeter of the nanotubes is *πd*, and when it is divided by the distance between two clusters (that is, 3.9 nm), we determine that first eight POM clusters form a ring, and these rings further self-assemble into nanotubes.

Interestingly, this SWNT formation approach can be even expanded to the system of clusters. POMs, which as discrete anionic clusters usually have sizes of ∼1 nm, are ideal ultrasmall building blocks for self-assembling and rolling. Nanorolls (Fig. 7a) were prepared under suitable conditions in a very similar solvent system to those of the above inorganic SWNTs. These rolled structures are indeed not multi-walled nanotubes, as the number of dark ridges under TEM is not equal in two sides of one roll. In this case, no short helical structures could be seen in the final products, because POM clusters cannot simply fuse together to form stable ionic bonds and recrystallize in a manner like other inorganic SWNTs. By increasing the reaction temperature or the reaction time, SWNTs could nevertheless be obtained from nanocoils like in other inorganic SWNT cases (Fig. 7b). More interestingly, no regular morphology could be seen in the transition between nanocoils and SWNTs (Supplementary Fig. 22). By roughly calculating the surface area of the nanorolls and SWNTs, we can find that they are on the same scale (Supplementary Fig. 23). Such observations enlighten us to think that SWNTs are formed via the adjustment of the structures of the rolls and transfer to SWNTs.

The detailed structures of the POM SWNTs and nanorolls were carefully studied by Fourier transform infrared spectroscopy (FT-infrared spectroscopy) and small angle X-ray diffraction (SAXRD; Fig. 7c,d, Supplementary Fig. 24). The FT-infrared results suggest that the oleylamine group is present in both the nanorolls and nanotubes. The SAXRD results show that the distances between the POM monomers are 3.8 nm in the nanorolls and 3.9 nm in SWNT. Considering the fact that the length of an oleylamine molecule is estimated to be ∼2 nm (ref. 37) and the size of one POM cluster is ∼1 nm, the proposed structures of nanoroll and SWNT are reasonable (Fig. 7e), by taking their alternation or bending into consideration. By further optimizing the reaction conditions, more cluster-based SWNTs can be expected.

## Discussion

In summary, the four types of SWNTs we prepared all possess remarkably uniform diameters. This may be due to the inherent properties of the ultrathin nanostructures and reacting medium. Once the nuclei/ultrathin nanostructures generated in the proper solution and immediately confined by the solvent/surfactant are determined, their interactions are determined and the folding and growth behaviours are thereby determined. Consequently, the SWNTs grow in the same way, leading to remarkably uniform diameters. Their good solubilities in non-polar solvents (Fig. 6d) promise the potential for post-treatment starting from dispersion. For example, the POM SWNT can be well aligned on substrate (Supplementary Fig. 25). The SWNTs are quite stable both in the solvent and as dried powders, and Co(OH)_2_ SWNTs showed good stability under electrochemical test (Supplementary Figs 26 and 27, Supplementary Methods).

The generalized synthesis method relies on the confinement of monomer size, in which the ultrathin building blocks enable the flexibility of the assembling structures, and tuning the environment can drive them to coil into SWNTs. Thus, we suspect that the flexible nature of the ultrathin structures that lead to the formation of SWNTs. To some extent, the ultrathin structure of the inorganic compound conjures the image of carbon chains or polymers, which may enable structures with more complexities and functions. The growth process described in this report not only provides insight into the crystal growth mechanism, but also steers the way for tube structure preparations, and it bridges the gap between carbon and other elements.

## Methods

### Synthesis of sulfide nanocoils and nanotubes

InAc_3_ (0.029 g) and 0.003 g S powder were dispersed in 8 ml octylamine in a 10-ml capacity Teflon-lined autoclave. The reaction is sensitive to the amount of each compound. Adding a little bit more S would lead to ultrathin nanobelt structures, while less S would result in nanocoils but not nanotubes. Nanocoils could be fabricated at 180 °C for 12 h, and nanotubes could be prepared at 220 °C for 24 h. The superlattice of the nanocoils was prepared using a similar method with a solvent composed of 1 ml ethanol and 7 ml octylamine. When cooled to room temperature, the products were collected and dispersed in cyclohexane with ultrasonication. The dispersion was washed by ethanol and after washing three times, the precipitate was dispersed in cyclohexane for further characterization. All types of nanotubes described in this report were washed in similar method.

### Synthesis of hydroxide nanotubes

SWNTs were synthesized as follows, 0.028 g CoSO_4_7H_2_O and 0.006 g trimethyltetradecylammonium chloride (TTAC) were added to a 10-ml capacity Teflon-lined autoclave, and 500 μl octylamine and 10 ml hexane were then added in sequence. The octylamine was stored at 40 °C and the precursors' colour in autoclave immediately changed to blue when octylamine was injected. After sonicating for several minutes, the autoclave was sealed and heated at 140 °C for 8 h. Double-walled nanotubes were achieved by using 0.028 g CoSO_4_7H_2_O, 0.024 g TTAC, 9 ml hexane and 1 ml ethanol (dissolve the TTAC in ethanol at first). Multilayer nanotubes were generated by increasing the amount of TTAC to 0.036 g. Adding even more TTAC would results in holes on wall.

### Synthesis of phosphate nanorings and nanotubes

Volume of 1 mol l^−1^ NiCl_2_ and KH_2_PO_4_ or NaH_2_PO_4_ aqueous solution were prepared first. For the nanotubes synthesis, 0.3 ml NiCl_2_ solution and 0.2 ml KH_2_PO_4_ were added into 6 ml EtOH in a 10-ml capacity Teflon-lined autoclave, and then 0.5 ml oleic acid and 2.5 ml oleylamine were injected. After heating at 150 °C for 8 h, the products were washed and dispersed in cyclohexane. For the nanoring synthesis, 0.3 ml NiCl_2_ solution and 0.2 ml NaH_2_PO_4_ were added into a mixture of 3 ml EtOH and 3 ml hexane, then 1 ml oleic acid and 2 ml oleylamine were injected. Then, the autoclave was heated at 150 °C for 8 h. The doping of Co^2+^ can be achieved by intruding CoCl_2_ solution into the system at the beginning of the reaction.

### Synthesis of polyoxometalate nanorolls and nanotubes

In a typical synthesis, 0.1 g of Na_2_H_5_P(W_2_O_7_)_6_ was dispersed in 4 ml ethanol in a 10-ml capacity Teflon-lined autoclave. Then, a mixture of 4 ml oleic acid and 1 ml oleylamine was added into the autoclave with vigorous stirring. After further stirring for 5 min, the autoclave was sealed and heated at 180 °C for 10 min or 70 °C for 3 h in an oven. When setting the reaction temperature at 180 °C, it would be better to immediately take the autoclave out of oven, otherwise the residual heat of oven may provide environment for the transit of nanorolls to nanotubes. The synthesis process of nanotubes was similar to the synthesis of the ultrathin nanorolls except that the conditions were 180 °C and 1 h.

### Autoacceleration

As for the slope of Fig. 5a, the amount of each precursor is the same in different batches of reaction with different reaction time, and the diameters of tubes are remarkably uniform, so we can determine that the amount of unreacted precursors by using: 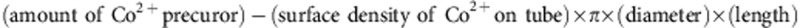 or (constant A)−(constant B) × (length). If we accept that the tube cannot grow at a relatively low temperature just by cooling (which is indeed true, that tube cannot grow under 110 °C, and it only takes about 10 min to decrease down to that temperature), then we may know that the absolute value of slope could indicate the consumption rate of the precursors. Consequently we can still infer the autoacceleration of reaction.

## Additional information

**How to cite this article:** Ni, B. *et al.* General synthesis of inorganic single-walled nanotubes. *Nat. Commun.* 6:8756 doi: 10.1038/ncomms9756 (2015).

## Supplementary Material

Supplementary Information Supplementary Figures 1-27, Supplementary Tables 1-2, Supplementary Methods and Supplementary References

## Figures and Tables

**Table 1:** The length of Co(OH)_2_ SWNT versus time.

**Time/h**	**Counts**	**Length/nm**	PDI*
1	1,000	76.4	1.67
1.5	1,000	93.4	1.30
2	1,000	127.8	1.77
2.5	1,000	129.0	1.76
3	1,000	165.0	1.74
3.5	1,000	149.3	1.55
4	1,000	200.7	1.34
4.5	1,000	277.7	1.48
5	1,000	321.6	1.47
5.5	1,000	355.3	1.43
6	1,000	353.2	1.46
8	1,000	407.8	1.28
10	1,000	401.5	1.42
12	1,000	513.3	1.59
96	234†	980.1	

PDI, polydispersity index; SWNT, single-wall nanotube.

^*^The average length is calculated as: 
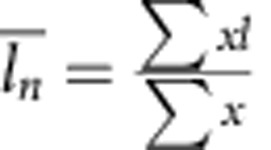
 weight average length: 
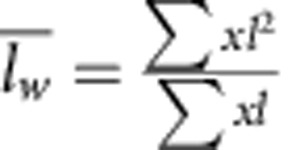
 and PDI: 
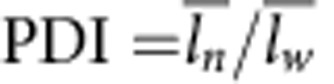
.

^†^For the specimen prepared at 96 h, only 234 nanotubes were measured because it was difficult to clearly identify 1,000 tubes in TEM images.
