# Supplementary material for: General synthesis of inorganic single-walled nanotubes
Source: Nat Commun. 2015 Oct 29;6:8756. doi: 10.1038/ncomms9756 (PMC4640082; doi:10.1038/ncomms9756)
Supplement: Supplementary Information — Supplementary Figures 1-27, Supplementary Tables 1-2, Supplementary Methods and Supplementary References [file ncomms9756-s1.pdf]

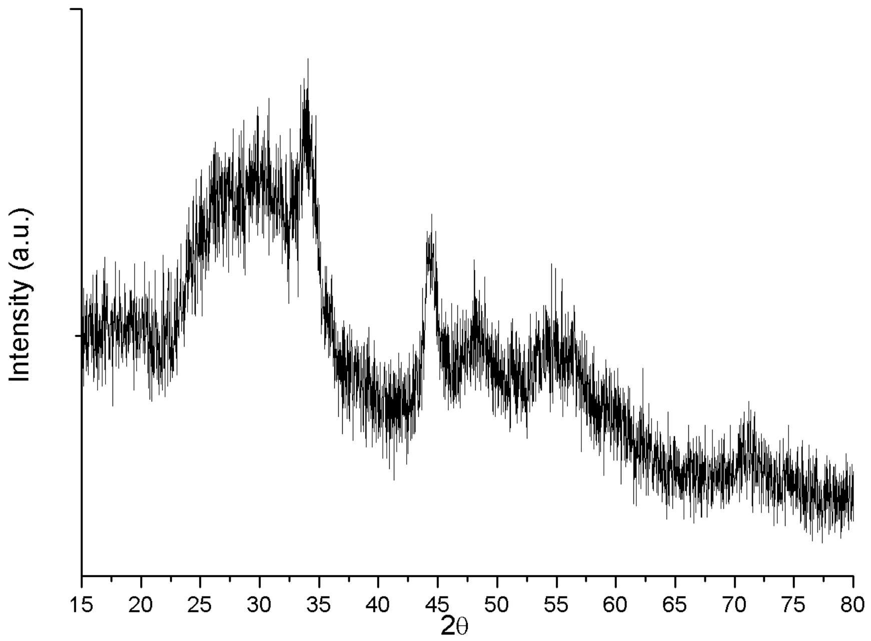

Supplementary Figure 1. XRD data of indium sulfide SWNT, the accurate positions of peaks are difficult to determine, this could be ascribed to the non-uniform lattice spacing with slight lattice bending as the result of the severe curvature. The shape of pattern is similar with our previous indium sulfide nanocoils.

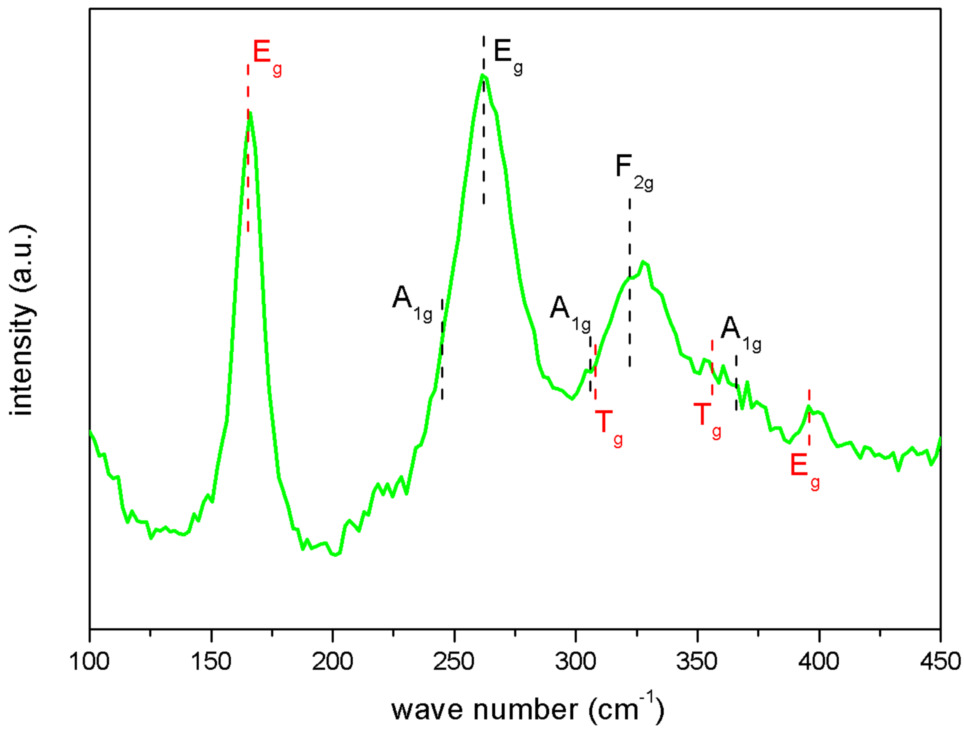

Supplementary Figure 2. Raman spectroscopy of indium sulfide. The black dash lines represent the signal's positions of In_2S_3[1] and the red dash lines represent the signals of In_2O_3[2]. The ultrathin nature of nanotube may lead to tendency of partial replacement of S by O existed in air or reaction media, leading to the co-existence of signals of In_2S_3 and In_2O_3.

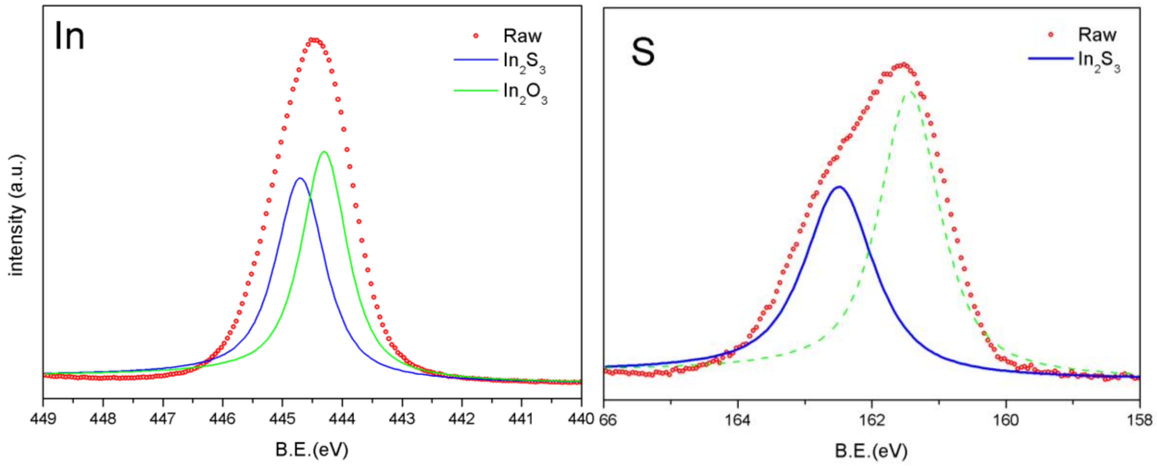

Supplementary Figure 3. The partial replacement of S by O can be further verified by X-ray photoelectron spectroscopy (XPS) of indium sulfide. The raw data of indium can be fitted by the $3d_{2/5}$ signals of indium in In_2S_3 and In_2O_3[3], suggesting indium atoms locates in different chemical environments. As for signals of S, the raw data vaguely shows a combination of two peaks, in which one at high binding energy (B.E.) could be indexed to In_2S_3[4]. However, the green dash peak cannot be explained as S or S_8 according to the database provided by National Institute of Standards and Technology. We may infer that the green dash peak is caused by the partial replacement of S by O in indium sulfide. Because the steric effect of O may lengthen In-S, thus leading to a lower B.E. of S comparing with pure In_2S_3.

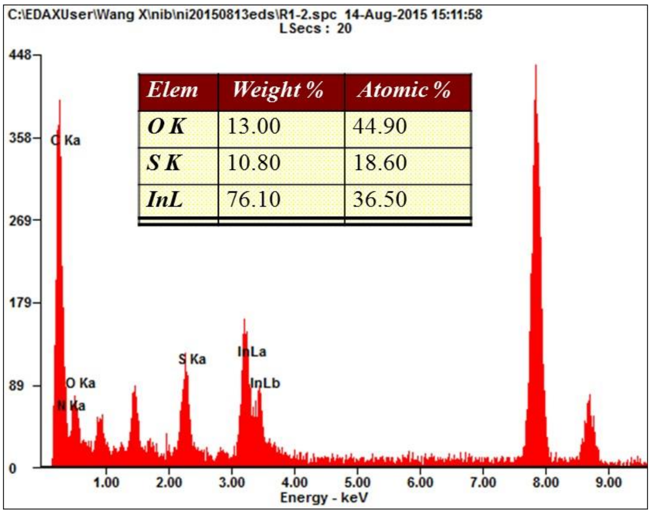

Supplementary Figure 4. Energy dispersive X-ray spectroscopy (EDS) and ICP results of indium sulfide. We selected more than 10 areas of indium sulfide SWNTs in TEM grid to measure their compositions, and the results were similar. The ratio of indium to sulfur is about 2:1. For conventional TEM grid may contain oxygen in their carbon membrane, so the ratio to oxygen is not accurate. EDS analysis is semiquantitative, so we used ICP to analysis the composition. ICP result indicated that the weight ratio of In:S = 60.45%:11.25%, corresponding to atomic ratio as In:S = 1.5:1. For ultrathin nanostructures (especially below 1 nm), they easily adsorb other species such as oxygen and have a large portion of surface which is easily to be partially oxidized, making it more complicated and difficult to characterize the precise elemental composition.

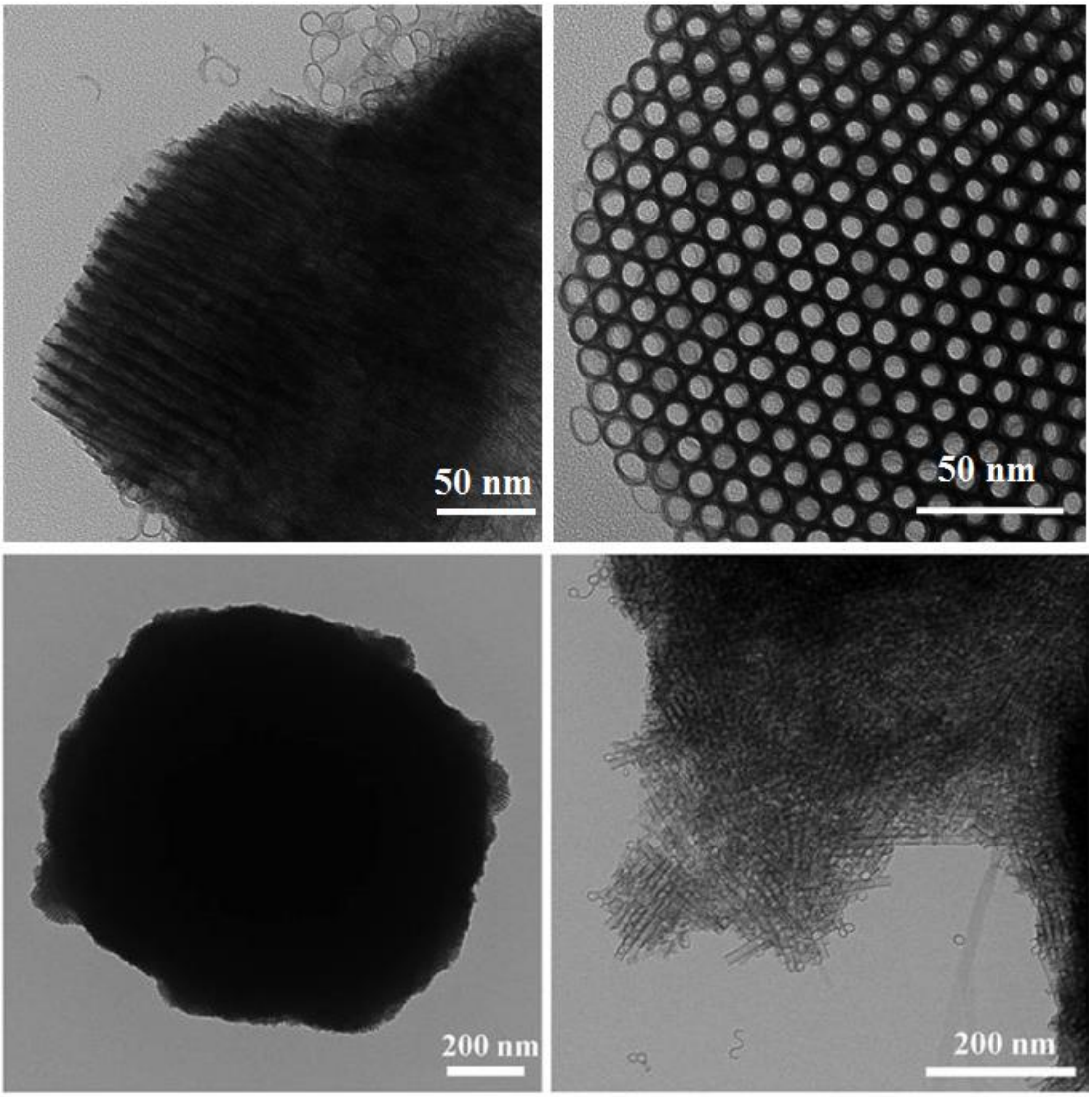

Supplementary Figure 5 Side view and top views of attached structures. The hollow channels could be seen as the on-going formation of tubes.

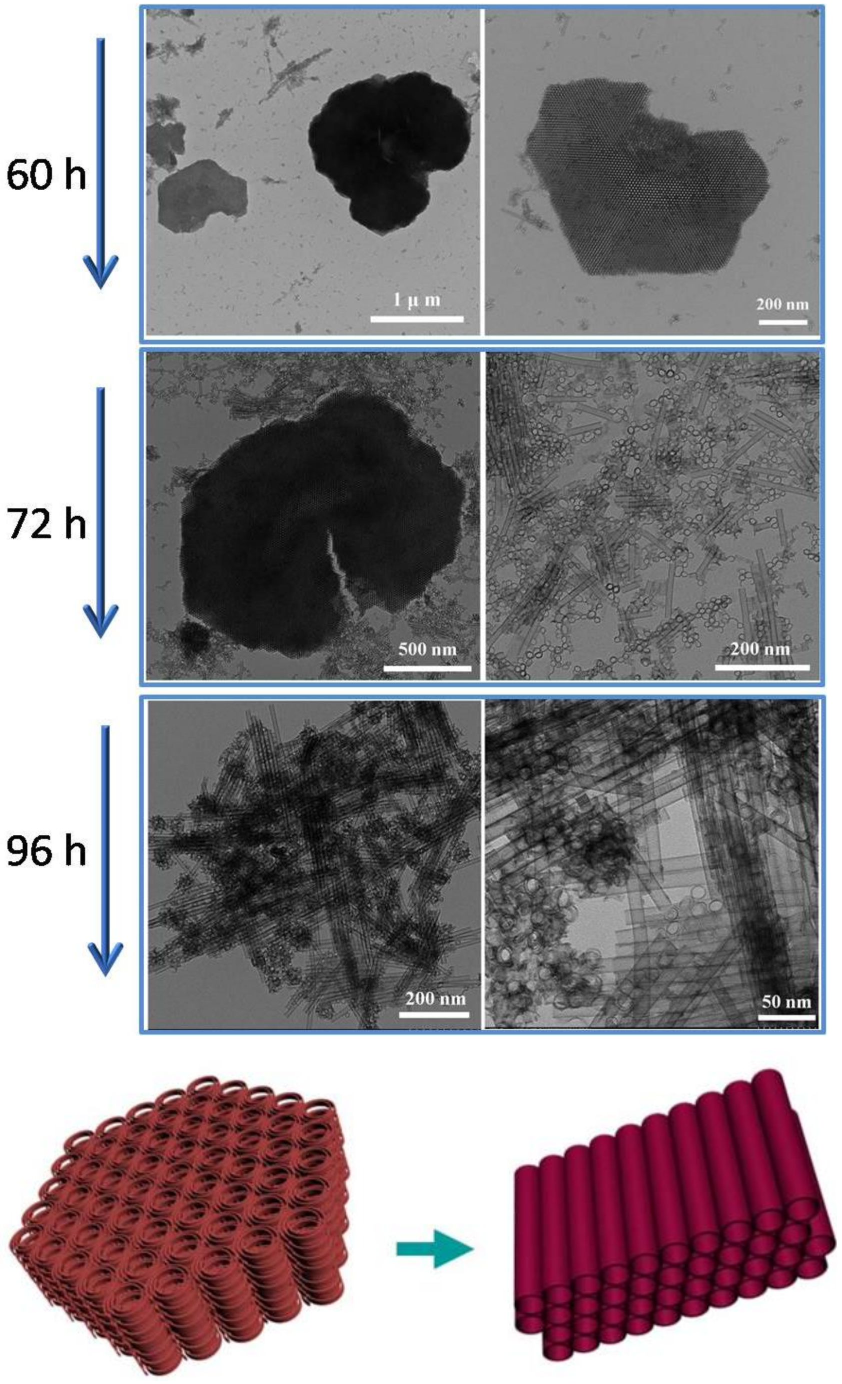

Supplementary Figure 6. As the reaction time increased, the nanocoil superlattice changed a lot, more and longer SWNTs can be prepared. Taking into account of the aforementioned hollow channels, the growth mechanism is drawn here.

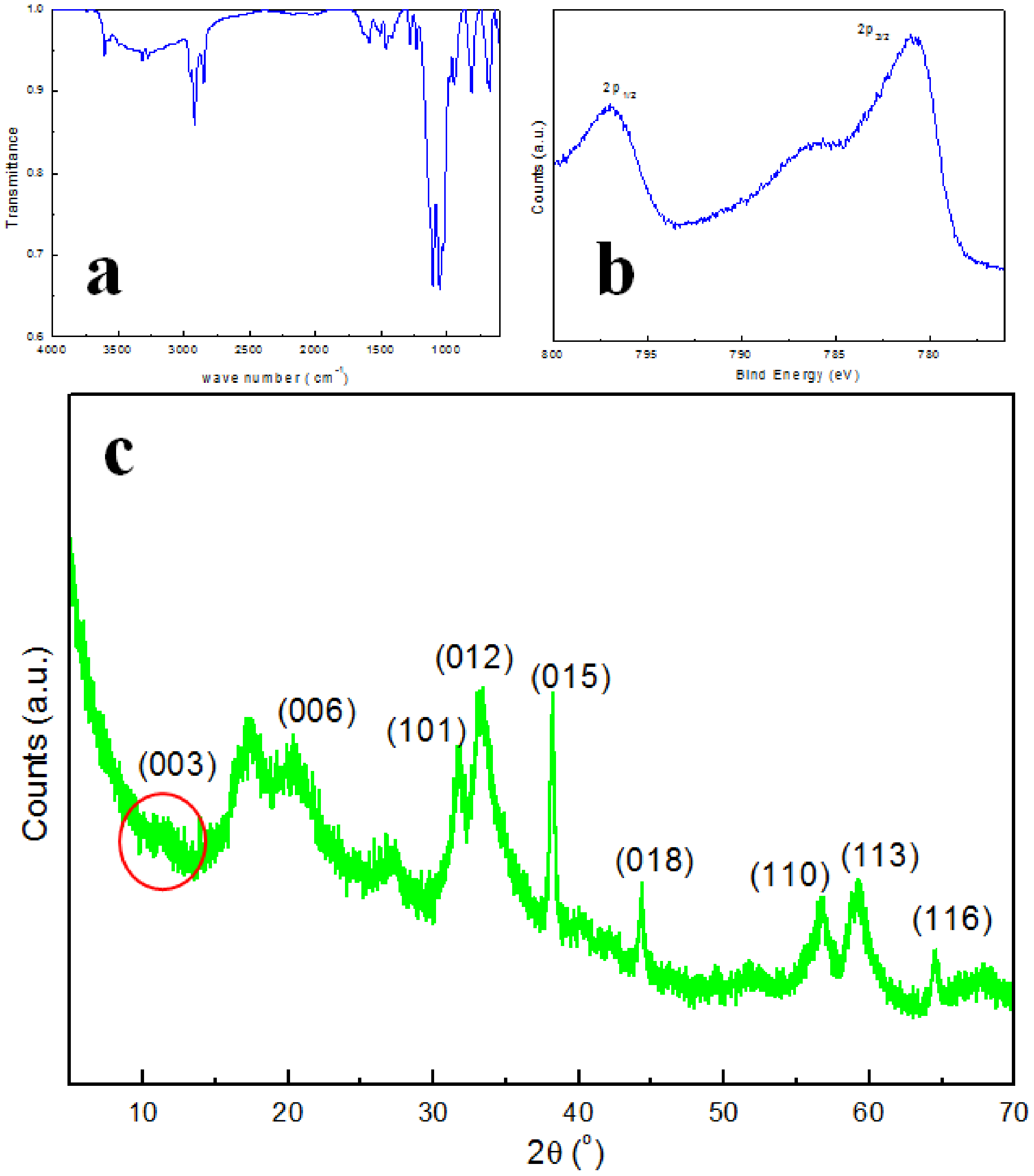

Supplementary Figure 7. Structure analysis of $Co(OH)_2$ SWNT. (a) FT-IR data, the strong absorption at 1040-1210 cm^{-1} could owe to SO_4^{2-}. The peaks (around 2931 cm^{-1} and 2848 cm^{-1}) correspond to the CH_2 and CH_3 stretching vibrations and the peaks at around 1510 and 1465 cm^{-1} correspond to the N−H mode, suggesting that amine molecules adsorb on SWNT. (b) The XPS peaks are identical with $Co(OH)_2$. (c) XRD peaks can be indexed into α-$Co(OH)_2$, but not β-$Co(OH)_2$. However, the (003) diffraction peak of α-$Co(OH)_2$, the strongest peak and characteristic of the layered structure, disappeared, meaning that the layered stacking of $Co(OH)_2$ was lost.

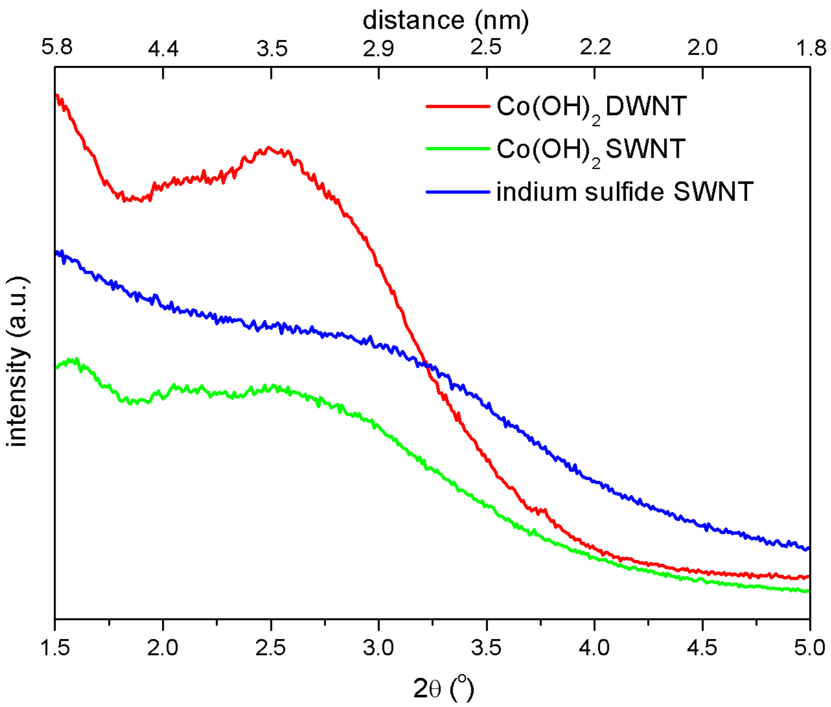

Supplementary Figure 8. Small angle XRD (SAXRD) data of indium sulfide SWNT, $Co(OH)_2$ SWNT and DWNT. Only $Co(OH)_2$ DWNT shows a broadening peak at distance of about 3.5 nm. Checking the TEM images of $Co(OH)_2$ DWNT we can find that the distance between different layers is about 3.5 nm, and the distances are not definitely the same everywhere, thus the peak is broadened. It's reasonable that $Co(OH)_2$ or indium sulfide SWNT doesn't feature layered structures, thus shows no peaks in SAXRD.

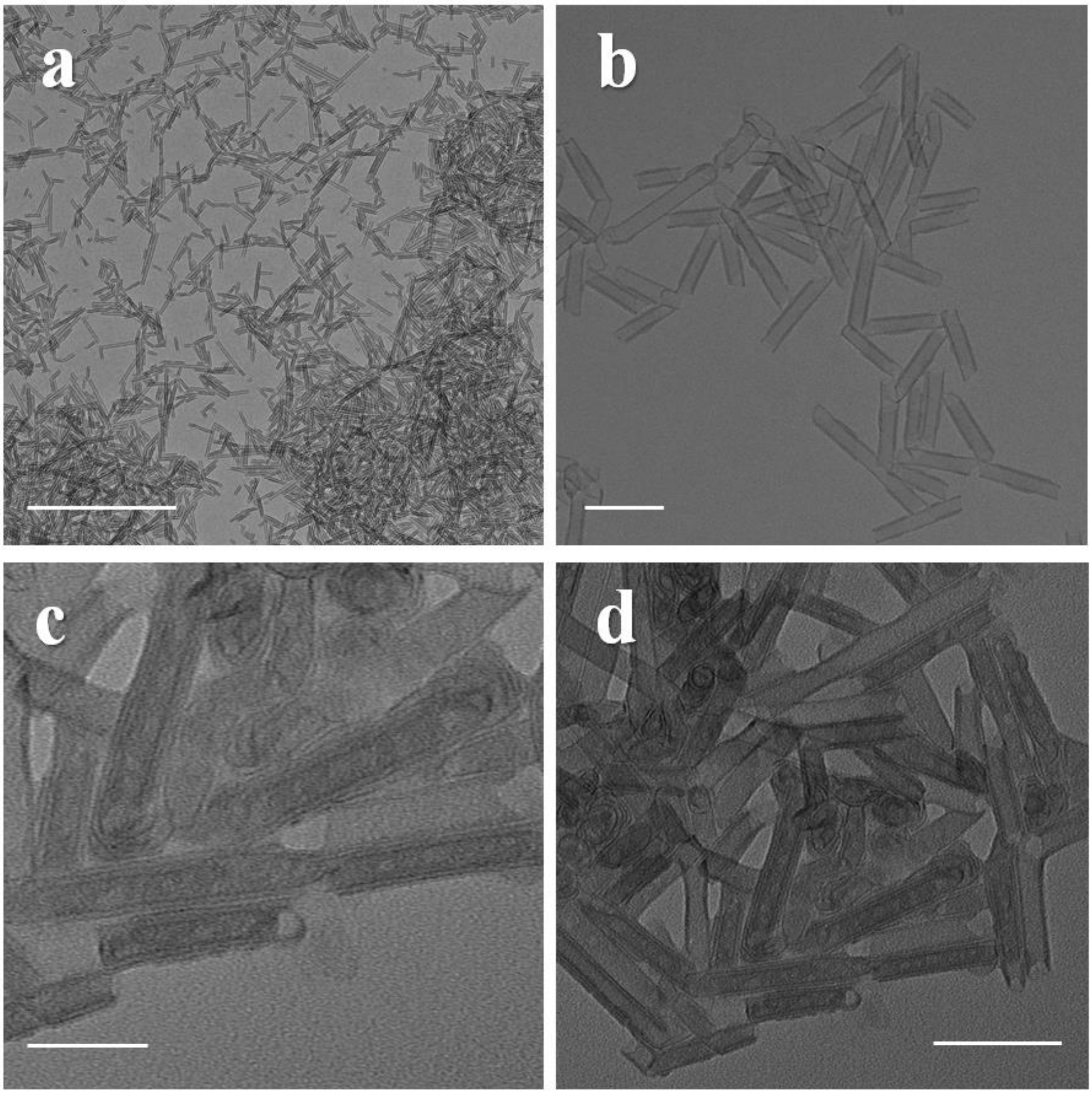

Supplementary Figure 9. Double layer nanotubes (a, b) and multi-layer $Co(OH)_2$ nanotubes (c, d). There are lots of holes on multi-layer nanotubes. This may be ascribed to that different layers possess different stabilities, thus layers with high energy would rupture. Scale bar: a,1μm; b, d, 100nm, c, 50nm.

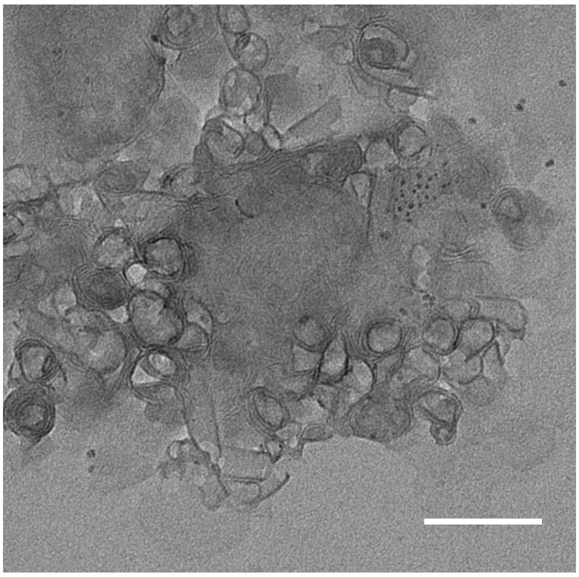

Supplementary Figure 10. In the case of $Co(OH)_2$ tube, when setting temperature at 110^oC, some sheets and spiral structures can be obtained, while no tube can be formed. Scale bar: 100nm.

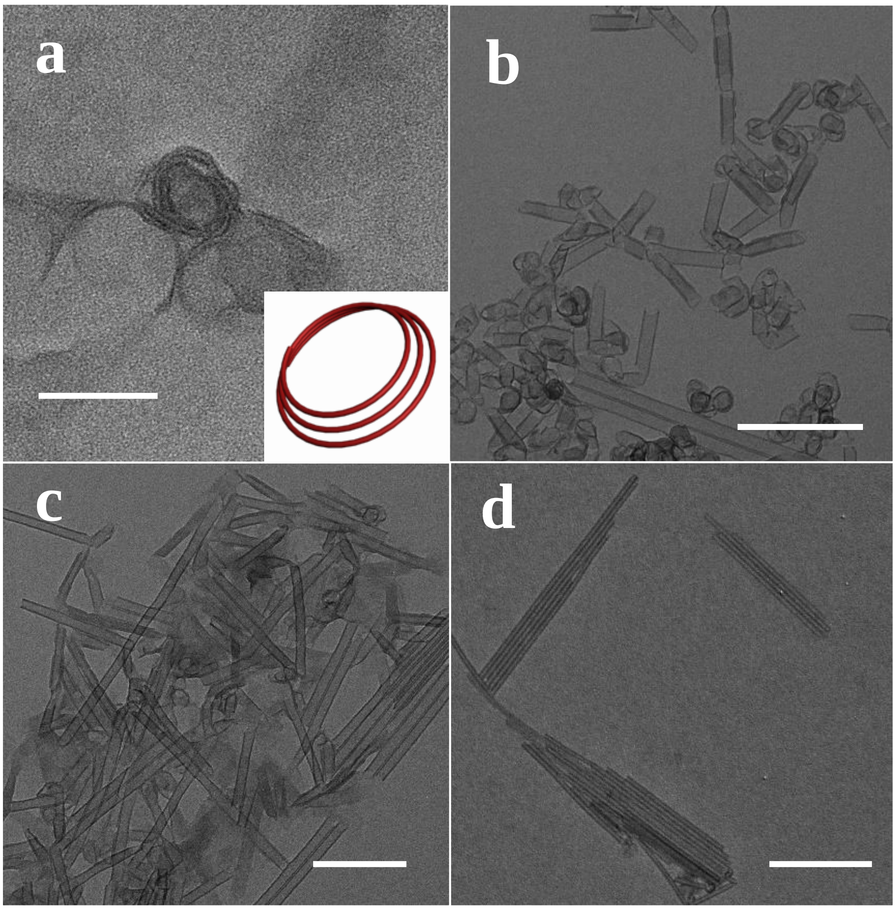

Supplementary Figure 11. TEM images of $Co(OH)_2$ SWNTs at different reaction time under unaffected cooling process, a: 15min, b: 45min; c: 4h; d: 12h. The morphology evolution goes from spiral structures to multiwall nanotubes and finally single wall nanotubes. The sheets at early stage may be due to the unreacted precursors. The products after 4h reaction time are all quite pure with only SWNTs in TEM images. Scale bar: a: 100nm; b, c, d, 200nm.

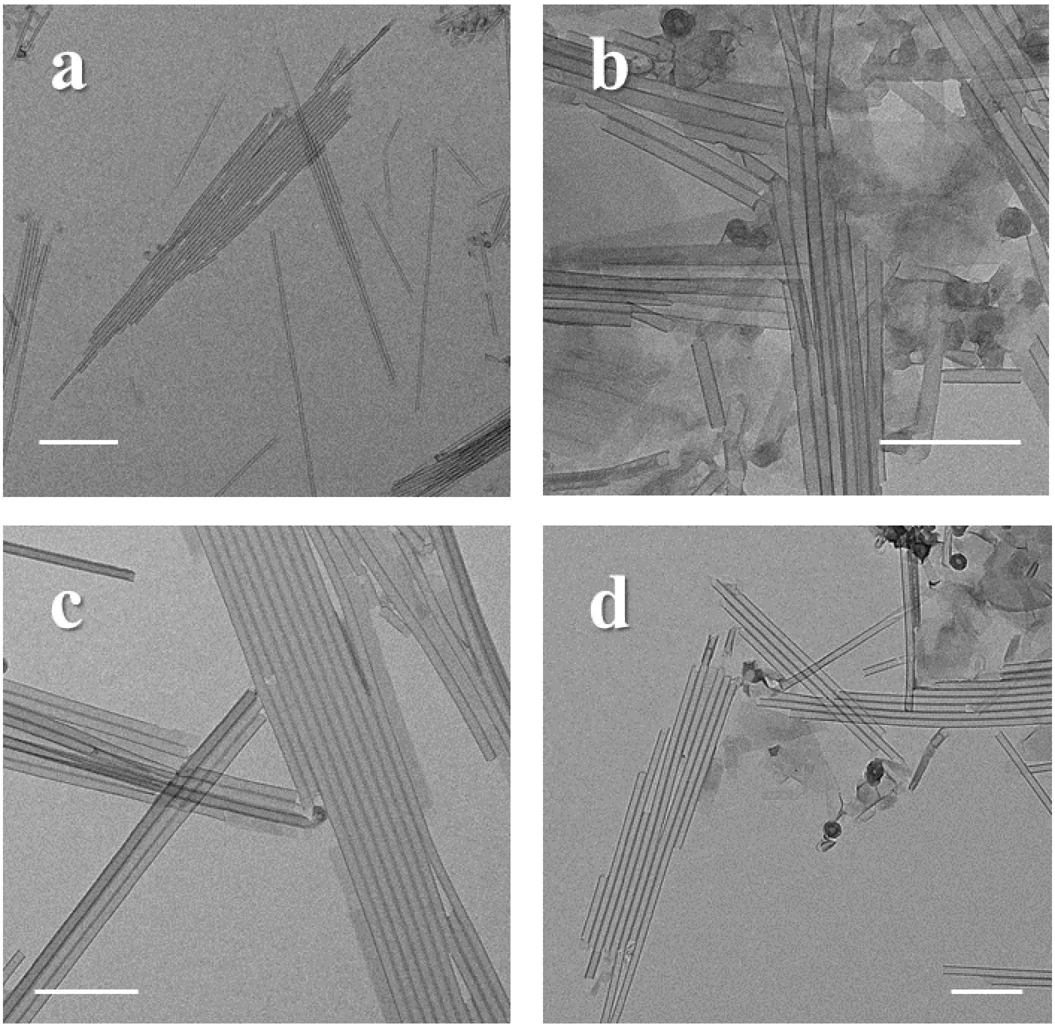

Supplementary Figure 12. When replace the hexane with octane(a), decane(b), dodecane(c) or tetradecane(d), SWNT can also be synthesized, and the diameters are almost the same. Data in other solvents are not shown. Reaction in solvent which possesses similar dielectric constant with hexane would give out such SWNT as products, the purity might be less. However, SWNT cannot be prepared in other solvent which shows different dielectric constant. Scale bar: a, 500nm; b, c, d, 200nm.

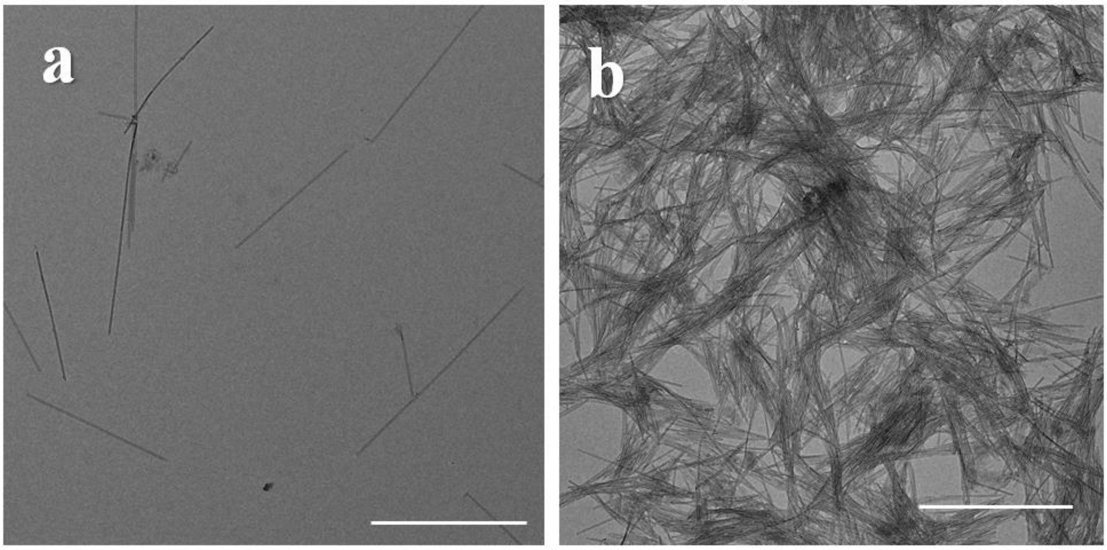

Supplementary Figure 13. TEM images of SWNTs at 96h, the products are quite pure, some bundles may be observed due to the interaction of adsorbed amines on the tubes. However, it’s not easy to clearly figure out 1000 tubes to calculate the average length. Scale bar: 2μm.

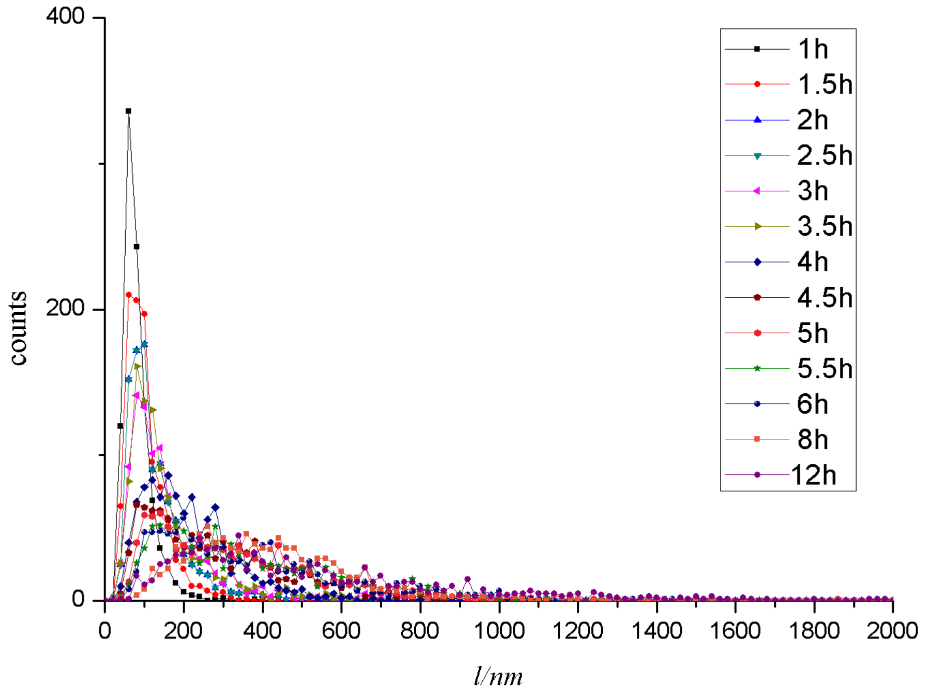

Supplementary Figure 14. Length distribution changes as reaction times increase. Although the diameter of tubes are remarkably uniform, their length distribution would become larger as reaction time goes. The broaden distribution resembles molecular weight distribution in polymer synthesis. (Bins: 20nm)

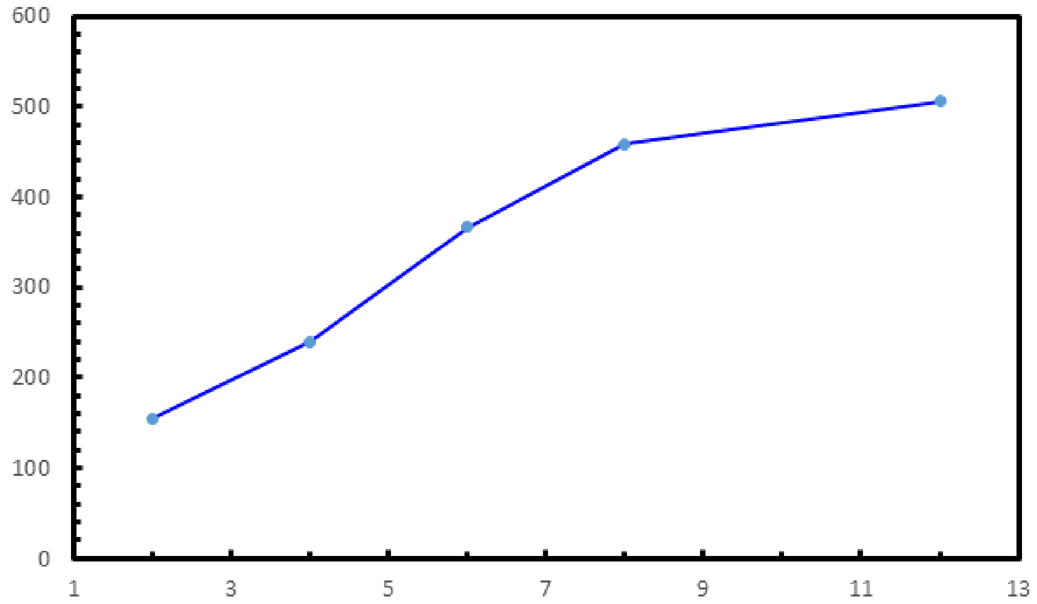

Supplementary Figure 15. Reproducibility of growth kinetics of $Co(OH)_2$ SWNT. The blue line which is draw by using black numbers in the Supplementary Table 1. It also shows a mild acceleration effect. The two sets of data were collected with a time interval of more than 8 months, however they are quite similar. It is worth mentioning that each group of synthesis is finished in the same day, as well as the measurement of length. In order to eliminate the error caused by selectively measuring, we measured every tube in a TEM image of low magnification, typically two or three TEM images may contain more than 1000 tubes.

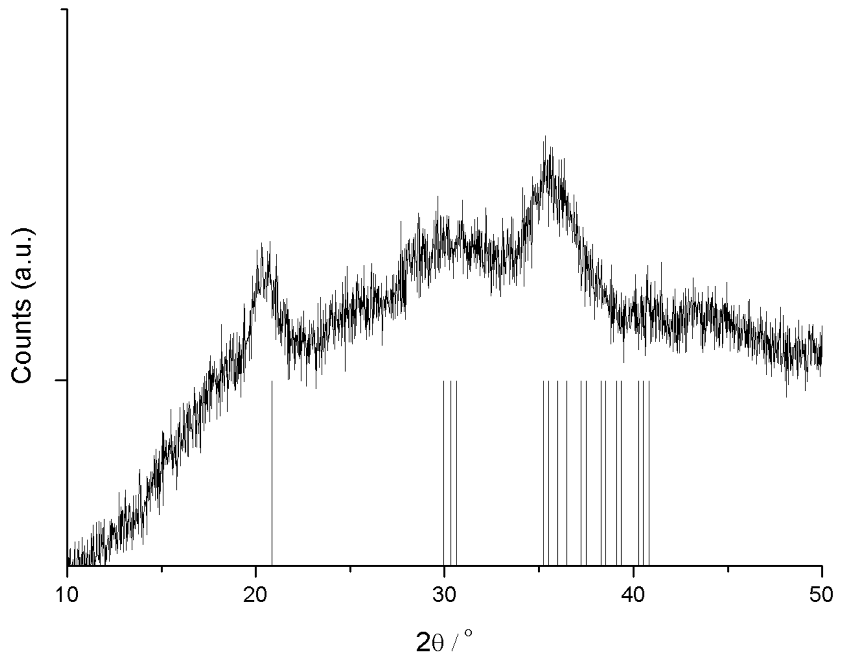

Supplementary Figure 16. XRD data of phosphate SWNT. The vertical lines indicate some peaks of $Ni_3(PO_4)_2$ (JCPDS: 38-1473), the heights of them doesn't relate their relative strength of different peaks. Although the peaks are broaden, several discernable peaks can fit with the $Ni_3(PO_4)_2$.

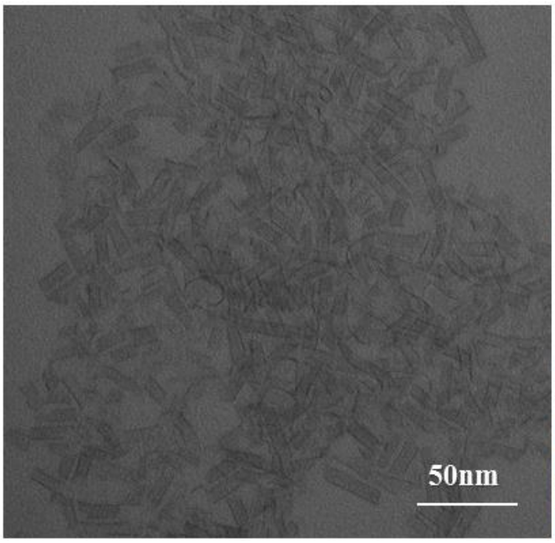

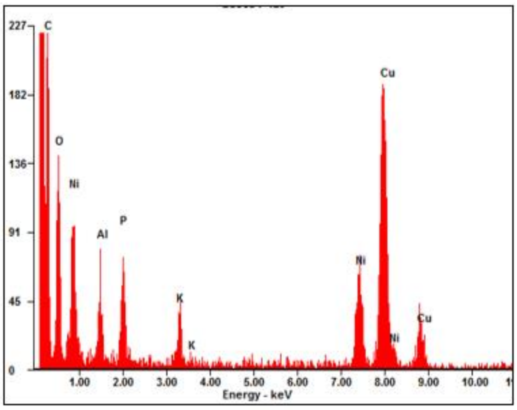

Elem	Weight %	Atomic %
P	25.30	36.80
K	15.30	17.60
Ni	59.40	45.60

Supplementary Figure 17. EDS analysis of $Ni_3(PO_4)_2$. By comparing the XRD data and the atomic ratio, we can conclude that signal of K may be the absorption of K salts.

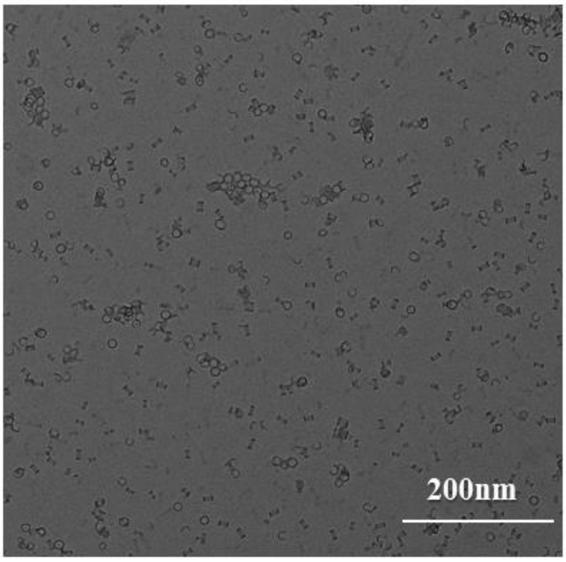

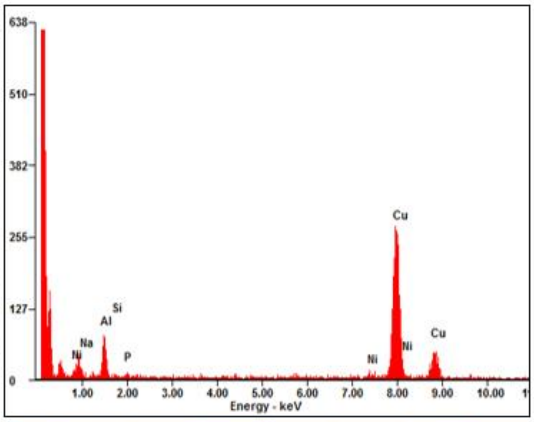

Elem	Weight %	Atomic %
NaK	23.70	38.00
P K	25.00	29.80
NiK	51.30	32.20

Supplementary Figure 18. EDS analysis of $Ni_3(PO_4)_2$. Similar with the phosphate SWNT case, the signal of Na may be absorption of Na salts.

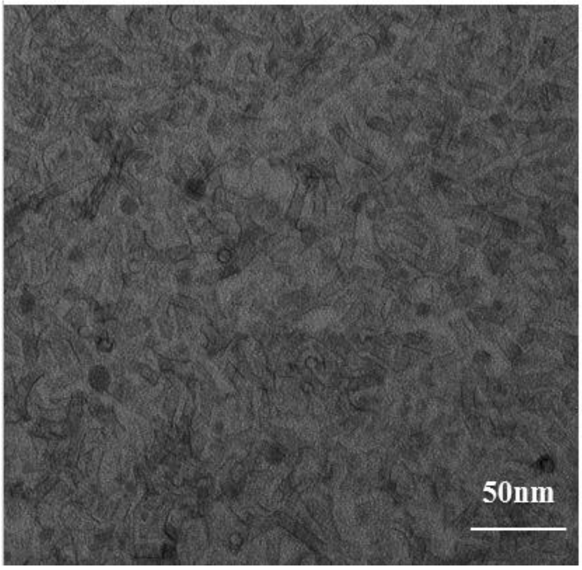

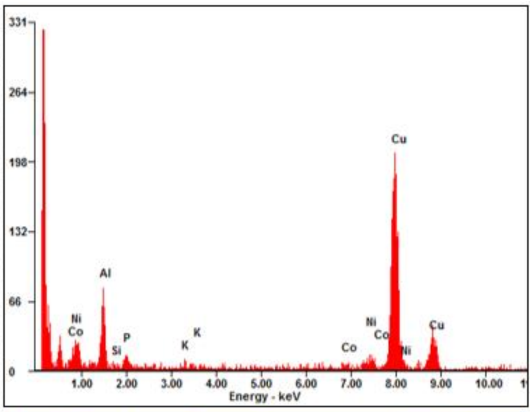

Elem	Weight %	Atomic %
P	23.00	34.90
K	08.70	10.40
Co	23.70	18.90
Ni	44.60	35.70

Supplementary Figure 19. Doping Co into $Ni_3(PO_4)_2$ SWNT. When introducing $CoCl_2$ (1/2 the amount of $NiCl_2$) at the beginning of reaction, the products show signal of Co. EDS analysis shows the ratio of Co:Ni in products is in consist with the precursors.

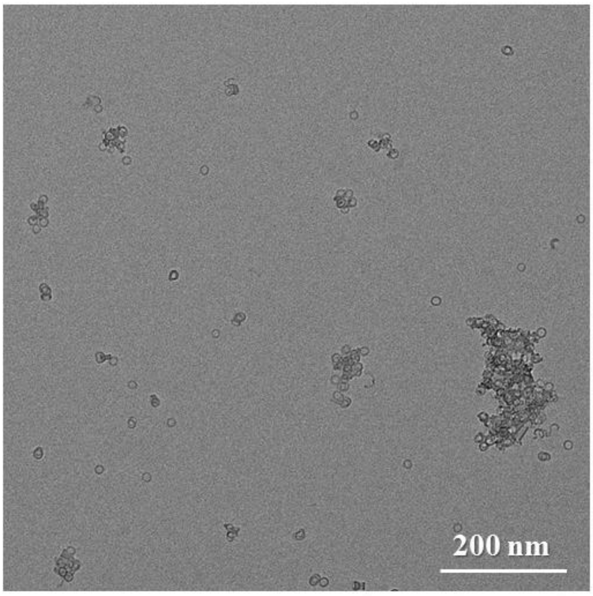

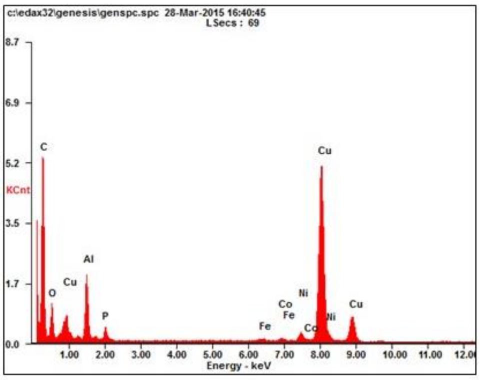

Elem	Weight %	Atomic %
Fe	17.40	18.10
Co	21.90	21.70
Ni	60.70	60.20

Supplementary Figure 20. Doping Fe and Co into $Ni_3(PO_4)_2$. By adding $Fe(NO_3)_3$ into the synthesis of phosphate nanorings, iron ions can be effectively incorporated into nanorings.

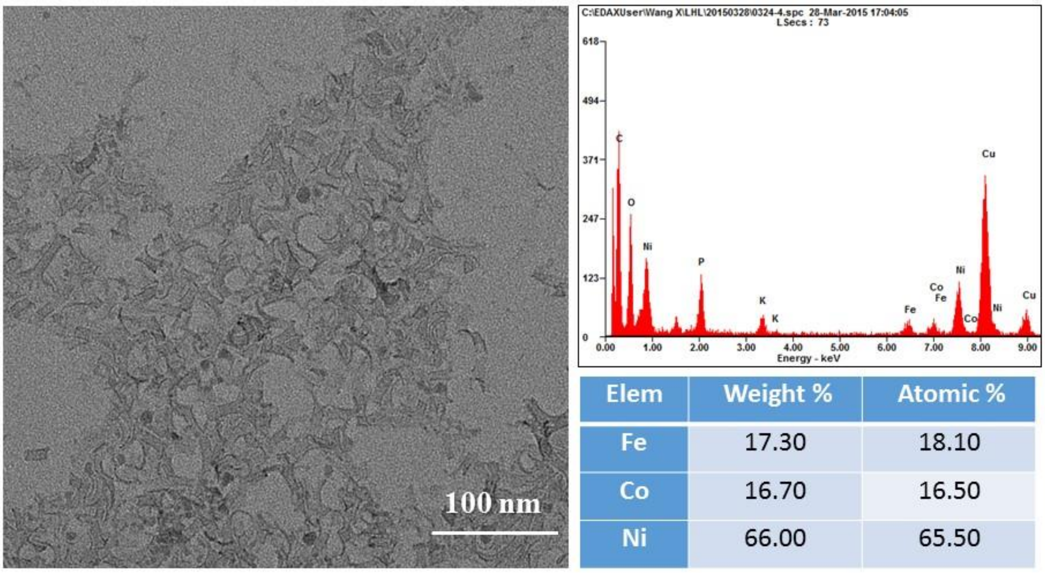

Elem	Weight %	Atomic %
Fe	17.30	18.10
Co	16.70	16.50
Ni	66.00	65.50

Supplementary Figure 21. Doping Fe into $Ni_3(PO_4)_2$. By adding $Fe(NO_3)_3$ into the synthesis of phosphate nanotubes, iron ions can be doped into nanotubes. Though the structure is changed, tubes can still been seen in the TEM image.

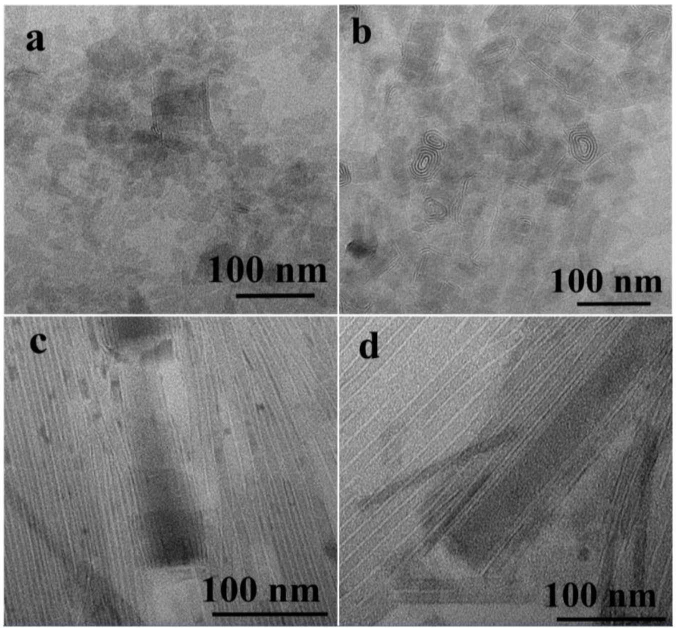

Supplementary Figure 22. TEM images of POM samples obtained at 20 (a), 30 (b), 40 (c) and 50 (d) min.

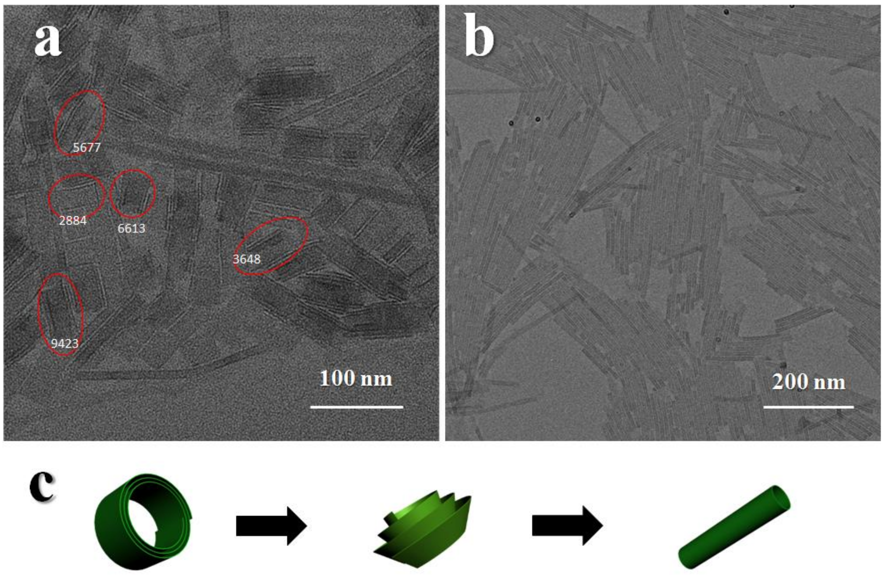

Supplementary Figure 23. Growth mechanism of POM SWNT. The number (unit: nm^2) in (a) indicate the surface area of nanorolls in the red circle nearby. The numbers are roughly calculated by considering the rolls as cylinders. If the ridges of two sides of one roll in TEM image are equal, for example, both are 2, we consider the surface area as $2\pi d$, here d is the mean diameter of cylinders. If the ridges of two sides of one roll in TEM image are not equal, for example, one side is 2 and the other side is 3, we consider the surface area as $2.5\pi d$, here d is the mean diameter of cylinders. We then consider the surface area of SWNTs in (b) by counting the average diameter and length of tubes, the surface area is calculated by πd, and found out that the value is 3254 nm^2. The surface area of rolls and tubes are at the same scale. Considering the error in calculating, we then proposed a mechanism (c) by self-adjusting and rolling of rolls, after healing of surface, the SWNT is formed.

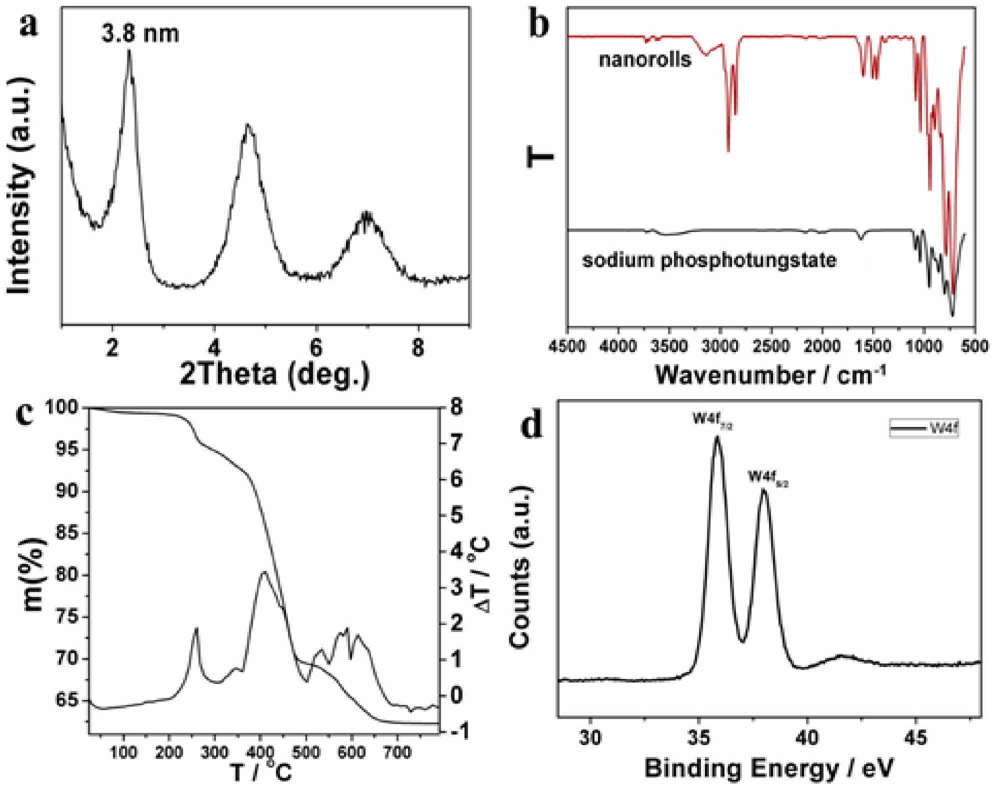

Supplementary Figure 24. Structure analysis of POM nanorolls. a) Small angle XRD (SAXRD); b) FT-IR; c) TGA; d) XPS of as-synthesized POM nanorolls. SAXRD data shows a distance at 3.8nm, this could be distance between POM particles. In FT-IR data, the peaks at 801 cm^{-1} and 855 $cm^{-}1$ are assigned to O-W-O antisymmetric stretching vibration modes of the bridging oxygen. The band at 953 cm^{-1} corresponds to W=O stretching mode of terminal oxygen. The band at 1083 cm^{-1} is ascribed to P-O stretching mode. TGA result shows that the sample begins to lose weight at 170 ^{o}C and stabilized since 650 ^{o}C due to combustion of oleylamine. XPS test exhibits two peaks at 35.5 and 37.7 eV which match reported values of W^{6+} oxidation state, corresponding to $W4f_{7/2}$ and $W4f_{5/2}$ states of W^{6+}

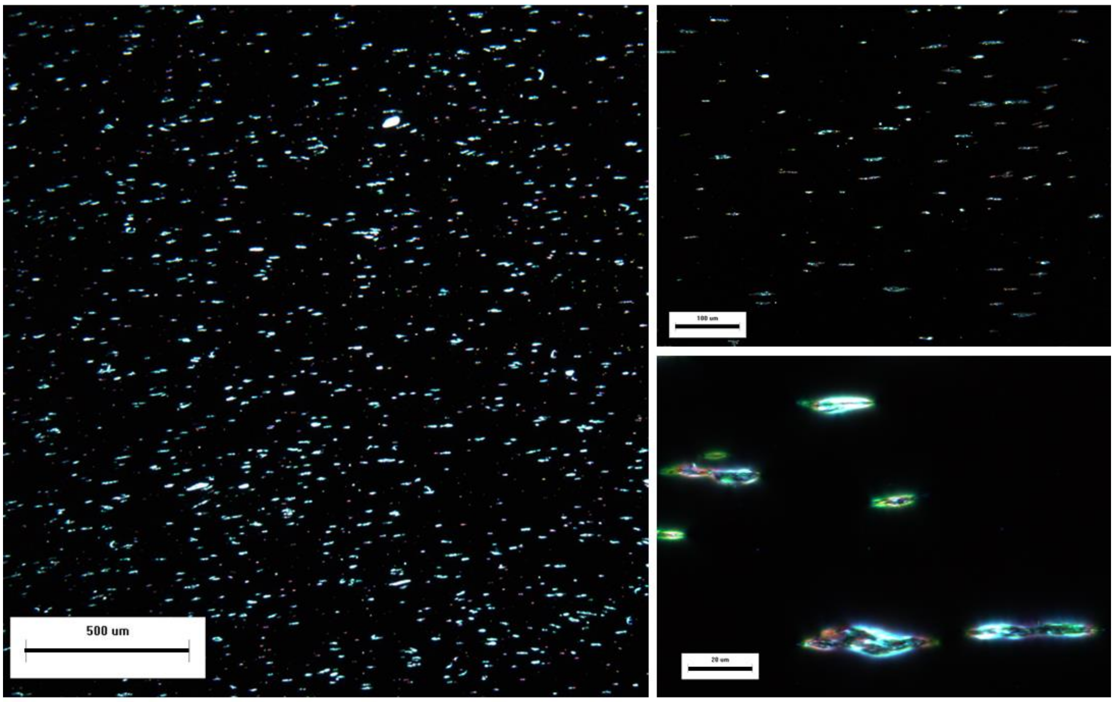

Supplementary Figure 25. Optic microscopy image of assembled POM SWNTs on silicon wafer, a bundle of SWNTs are aligned in the same direction.

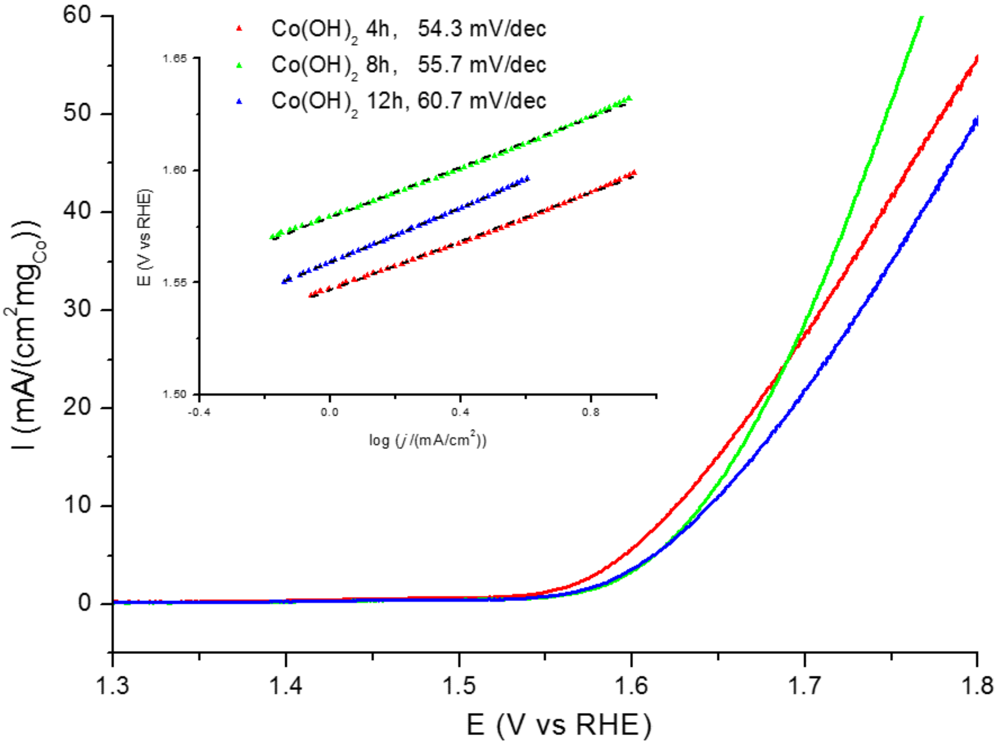

Supplementary Figure 26. Polarization curves and Tafel plots (inset) of $Co(OH)_2$ SWNTs prepared under different reaction times. Red line stands for products with reaction time of 4h, green line for 8h and blue line for 12h, the length of them are different. However, the performances are similar when current densities are reduced to masses of metal. The slopes of Tafel plots are similar and their values are displayed in the graph. Black dark lines indicate the fit lines. Phosphate SWNTs with doping or not were also test for OER, data are not shown here.

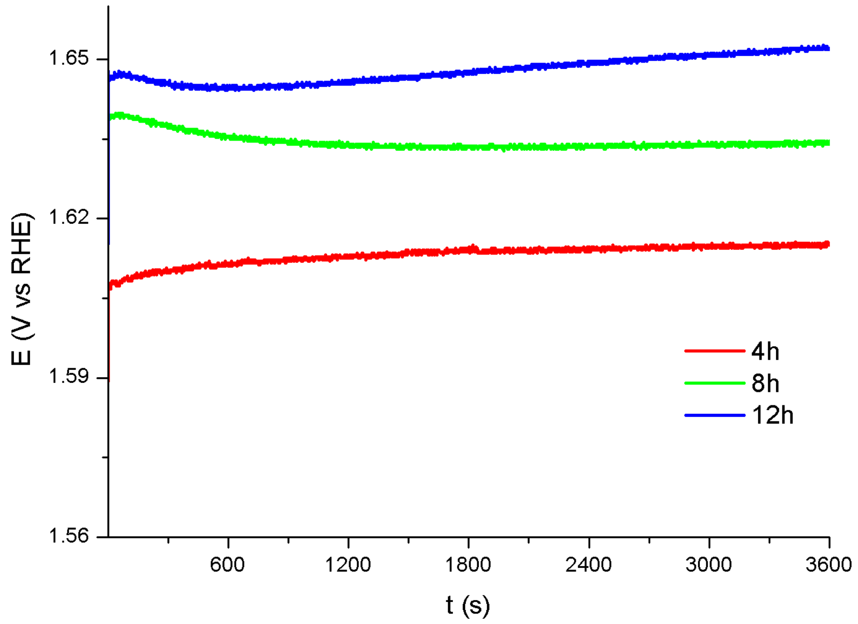

Supplementary Figure 27. Electrochemical stabilities of $Co(OH)_2$ SWNTs prepared under different reaction times. The tests were achieved by applying constant current density ($10mA/cm^2$). The results indicate that $Co(OH)_2$ SWNTs are stable for electrochemical test. Red line stands for products with reaction time of 4h, green line for 8h and blue line for 12h.

Supplementary Table 1. Reproducibility of tube length and their polydispersity index*

Time (hours)	Counts	Length (nm)	PDI	Length (nm) (old)	PDI (old)
2	1000	154.4	1.76	127.8	1.77
4	1000	268.9	1.33	200.7	1.34
6	1000	366.8	1.36	353.2	1.46
8	1000	458.8	1.26	407.8	1.28
12	1000	506.2	1.49	513.3	1.59

***** The red words and numbers in the table represent the set of data that we show in Table 1 in main text of this research (denote as old), and the black words and numbers are another set of data.

Supplementary Table 2. Tube length of unaffected cooling*[a] and fast cooling*[b]

Time (h)	Unaffected cooling (nm)	Fast cooling (nm)
4	200.74	122.99
6	353.23	238.80
8	407.75	320.41
10	401.48	320.48

*a unaffected cooling means naturally cool down the autoclave in air, typically it would take about 1.5h for the autoclave to totally cool down to room temperature. However, it would only take less than 20 min for the autoclave to decrease below 110 ^{o}C, under which temperature the tube cannot form.

*b fast cooling means that directly flush the autoclave with cold water, the time for totally cooling down to room temperature is typically less than 10 min.

Supplementary Methods: In a typical sample preparation of oxygen evolution reaction test of $Co(OH)_2$ SWNT, after washing and dispersing the $Co(OH)_2$ SWNT in 2mL cyclohexane, 1mL cyclohexane solution was transferred into PE tube, then 0.05mL nafion was injected and 1mg XC-72 carbon was added, and sonicate for 30mins. Then 6μL solution was dropped on the glassy carbon electrode with a surface area of 0.196cm^2. The amounts of $Co(OH)_2$ were determined by inductively coupled plasma optical emission spectrometer (ICP-OES), and found out the concentration of Co^{2+} in solution was about 2.2mg/mL.

The oxygen evolution reaction performance were test in KOH (1mol/L) and conducted with Princeton P4000 electrochemical workstation using a three electrode cell where Pt foil served as the counter electrode. Cyclic voltammetry (CV) were used to stabilize the materials. After the CV results were stable, set the rotation speed of electrode at 1600 rpm and measure the linear sweep voltammetry (LSV) at scan rate of 10mV/s. After LSV test, Tafel plots were got by the Tafel test function of Princeton P4000. The chronopotential tests were used to study the stabilities of products, set the current at 1.96mA, correspond to 10mA/cm^2, and test for 1 hour.

Supplementary References:

1 Tao, H. Z., Mao, S., Dong, G. P., Xiao, H. Y. & Zhao, X. J. Raman scattering studies of the Ge-In sulfide glasses. *Solid State Communications* **137**, 408-412, doi:10.1016/j.ssc.2005.12.032 (2006).

2 Garcia-Domene, B. *et al.* High-pressure lattice dynamical study of bulk and nanocrystalline In2O3. *J. Appl. Phys.* **112**, 123511, doi: 10.1063/1.4769747 (2012).

3 Briggs, D. *Handbook of x-ray and ultraviolet photoelectron spectroscopy*. (Heyden, 1977).

4 Battistoni, C., Gastaldi, L., Lapiccirella, A., Mattogno, G. & Viticoli, S. Octahedral vs tetrahedral coordination of the co(II) ion in layer compounds: CoxZn1−xIn2S4(O⩽x⩽0.46) solid solution. *J. Phys. Chem. Solids* **47**, 899-903, doi: 10.1016/0022-3697(86)90062-4 (1986).
